# A Dynamic Multi-Niche Biogeography-Based Optimization Algorithm and Its Application to Robot Path Planning

**DOI:** 10.3390/biomimetics11030221

**Published:** 2026-03-19

**Authors:** Xiaojie Tang, Pengju Qu, Zhengyang He, Chengfen Jia, Qian Zhang

**Affiliations:** 1 School of Intelligent Manufacturing, Sichuan University Jinjiang College, Meishan 620860, China; hezhengyang@scujj.edu.cn (Z.H.); jiachengfen@scujj.edu.cn (C.J.); zhangqian@scujj.edu.cn (Q.Z.); 2 Key Laboratory of Advanced Manufacturing Technology of the Ministry of Education, Guizhou University, Guiyang 550025, China; 20140497@git.edu.cn; 3 Engineering Training Center, Guizhou Institute of Technology, Guiyang 550003, China

**Keywords:** biogeography-based optimization, path planning, metaheuristic, dual-source migration mechanism, hybrid elite preservation

## Abstract

Biogeography-based optimization (BBO) is a population-based metaheuristic algorithm inspired by species migration among habitats. However, the original BBO often suffers from premature convergence and insufficient population diversity when solving complex optimization problems. To address these limitations, this paper proposes a novel dynamic multi-niche biogeography-based optimization (DMBBO) algorithm. DMBBO incorporates three effective strategies: a dynamic multi-niche population structure to maintain diversity and enhance parallel search capability, a dual-source migration mechanism to improve information exchange efficiency, and a niche-level hybrid elite preservation strategy to stabilize convergence behavior and improve solution quality. Extensive experiments were conducted on the CEC2022, CEC2020, and CEC2019 benchmark test suites under different dimensional settings. The experimental results demonstrated that DMBBO consistently outperformed 23 state-of-the-art algorithms in terms of optimization accuracy, convergence speed, and robustness, with statistically significant improvements validated by Friedman ranking and Wilcoxon rank-sum tests. An ablation study and convergence behavior analysis further confirmed the effectiveness of the proposed strategies. Additionally, DMBBO was applied to robotic path planning problems in grid-based environments involving six different scenarios with varying map sizes and obstacle densities. The results showed that DMBBO is capable of generating shorter and more stable paths in both simple and complex environments, highlighting its strong applicability to constrained optimization problems.

## 1. Introduction

Metaheuristic optimization algorithms have been widely applied to solve complex optimization problems due to their flexibility, derivative-free nature, and strong global search capability. Such problems are often characterized by high dimensionality, nonlinearity, multimodality, and complex constraints, making them difficult to address using traditional deterministic or gradient-based optimization methods [1,2]. As a result, population-based metaheuristic algorithms inspired by natural phenomena have attracted increasing attention in recent decades.

Many of these methods are developed within the framework of biomimetics. Biomimetics studies biological behaviors, evolutionary mechanisms, and ecological processes in nature and applies these principles to computational model design. By imitating the adaptability and collective intelligence of biological systems, biomimetic optimization algorithms provide effective tools for solving complex optimization problems.

Among various metaheuristic approaches, swarm intelligence and evolutionary algorithms, such as particle swarm optimization (PSO) [3], genetic algorithms (GAs) [4], differential evolution (DE) [5], firefly algorithms (FAs) [6], gray wolf optimizer (GWO) [7], moth-flame optimization algorithm (MFO) [8], whale optimization algorithm (WOA) [9], and other nature-inspired methods [10,11,12,13], have demonstrated promising performance in diverse application domains. Khatir et al. [14] proposed a hybrid algorithm combining particle swarm optimization (PSO) with the YUKI algorithm, applying it to the dual-crack detection of carbon fiber-reinforced polymer (CFRP) cantilever beams. Khishe et al. [15] employed a swarm-based hierarchical differential evolution (HPDE) for optimizing model parameters of proton exchange membrane fuel cells (PEMFC). Results demonstrated HPDE’s capability to accurately and rapidly extract PEMFC model parameters. Zhang et al. [16] proposed the elite-driven gray wolf optimizer (EDGWO) algorithm for feature selection in medical datasets. To optimize deflection prediction using mechanical parameters as input data, Rahmani et al. [17] integrated a deep neural network (DNN) with a novel enhanced whale optimization algorithm (EWOA). Experiments demonstrated that this hybrid framework outperformed traditional optimization-based models.

Robot path planning, a key research topic in autonomous navigation, aims to determine the optimal or near-optimal path from a starting position to a target location while avoiding obstacles and satisfying environmental constraints. Traditional path planning methods include graph-theoretic such as Dijkstra’s algorithm (1959) [18], A-star (1968) [19], and sampling-based methods like rapidly-exploring random trees (RRTs, 1996) [20]. These approaches may encounter issues of high computational cost or limited adaptability in complex environments. Many meta-heuristic optimization algorithms and their variants, however, are widely applied in path planning due to their robust global search capabilities and flexibility. Ahmad et al. [21] proposed the alpha–beta guided particle swarm optimization (ABGPSO) algorithm for mobile robot path planning, demonstrating its superiority over other algorithms in static experimental environments. Zhang et al. [22] combined ant colony optimization (ACO) and a genetic algorithm (GA) to solve multi-map path planning problems in mine disaster rescue scenarios. Zhang et al. [23] proposed an enhanced dung beetle optimization (EDBO) algorithm incorporating a search radius-based node selection strategy and applied it to path planning. Zhao et al. [24] introduced a mutation-based gray wolf optimizer (MYIGWO) integrating a dual mutation strategy for path planning tasks. Results demonstrated MYIGWO’s significant performance advantages in experiments.

However, no single algorithm can perform best on all optimization problems. This fact is explained by the no-free-lunch theorem [25,26]. Therefore, improving existing algorithms or designing better variants for specific problem types is still an important research topic.

Biogeography-based optimization (BBO) [27], proposed by Simon, is a population-based evolutionary algorithm inspired by the migration of species between habitats. In BBO, candidate solutions are treated as habitats, and solution features are exchanged through migration based on immigration and emigration rates. Because of its simple structure and clear concept, BBO has been applied to many optimization problems, such as engineering design, scheduling, and control. Chen et al. [28] combined the differential evolution algorithm with BBO and proposed the HBBO algorithm to solve the three-dimensional packing problem (3D-BPP). Du et al. [29] used an improved BBO algorithm to solve the flow shop scheduling problem (FSSP), and the results showed better performance than other methods. Kaveh et al. [30] proposed a three-dimensional migration model based on BBO (TDMBBO) to solve the constrained linear p-median problem in ambulance station planning. Zhao et al. [31] proposed a hybrid BBO algorithm with migration and mutation (BBOHMM) to reduce the side lobe level (SLL) in antenna array optimization problems. Experimental results showed that BBOHMM achieved lower SLL than other BBO variants and swarm intelligence algorithms. However, many studies have shown that the original BBO algorithm still has some limitations. These include fast loss of population diversity, early convergence, and poor balance between global search and local search, especially for high-dimensional or complex multimodal problems [32,33,34].

To overcome these drawbacks, various enhancement strategies have been proposed in the literature. These improvements can generally be categorized into several groups [35,36,37,38]. First, hybridization strategies combine BBO with other optimization techniques, such as differential evolution or genetic operators, to enhance exploration capability. Second, adaptive parameter control mechanisms dynamically adjust migration or mutation parameters to improve search efficiency. Third, population structure optimization strategies, including multi-population or niche-based mechanisms, aim to maintain diversity and prevent premature convergence.

Although these approaches have improved the performance of BBO to some extent, many existing variants still rely on single-population structures or fixed migration patterns, which may limit their ability to effectively balance exploration and exploitation in complex optimization landscapes. In particular, maintaining population diversity while ensuring efficient information exchange remains a challenging issue in BBO-based optimization.

Motivated by the above observations, this paper proposes a novel dynamic multi-niche biogeography-based optimization (DMBBO) algorithm. The proposed method introduces three complementary strategies to address the limitations of the original BBO: (1) a dynamic multi-niche population structure that partitions the population into multiple niches and adaptively adjusts their composition to preserve diversity and enhance parallel search capability; (2) a dual-source migration mechanism that enables more flexible and effective information exchange while preventing excessive homogenization of solutions; and (3) a niche-level hybrid elite preservation strategy that retains high-quality solutions within and across niches to improve convergence stability and optimization accuracy. These three mechanisms operate at distinct levels of the evolutionary process—population structure, information migration, and elite selection—forming a comprehensive optimization framework. Consequently, compared to existing BBO variants, the proposed DMBBO achieves a more effective balance between maintaining population diversity, enhancing information exchange efficiency, and coordinating exploration–exploitation trade-offs. It demonstrates superior optimization accuracy, robustness, and convergence properties.

To fully evaluate the performance of the proposed DMBBO algorithm, extensive experiments were carried out on the CEC2022, CEC2020, and CEC2019 benchmark test suites with different problem dimensions. The experiments included analysis of convergence behavior, statistical tests, and ablation study to verify the effectiveness of each proposed strategy. In addition, DMBBO was applied to robot path planning problems in grid-based environments with different map sizes and obstacle densities. This was done to show its practical use in real-world constrained optimization problems.

The main contributions of this paper are summarized as follows.

- A new dynamic multi-niche biogeographic optimization algorithm (DMBBO) is proposed. The algorithm introduces three effective improvement strategies into the BBO framework: a dynamic multi-niche population structure, a dual-source migration mechanism, and a niche-level hybrid elite retention strategy. These strategies help reduce the loss of population diversity and avoid premature convergence in the original BBO algorithm.
- An ablation experiment is conducted to verify the effectiveness of each proposed strategy. Convergence behavior analysis further shows that DMBBO has stronger global search ability and a more stable optimization process.
- Extensive experiments on the CEC2022, CEC2020, and CEC2019 benchmark test suites show that DMBBO achieves better performance than other algorithms in solution quality, convergence speed, and robustness.
- The effectiveness of DMBBO is further validated through robot path planning simulations across six simple and complex environments.

The remainder of this paper is organized as follows. Section 2 introduces the fundamental principles of the original BBO algorithm. Section 3 presents the proposed DMBBO algorithm in detail. Section 4 reports the benchmark experimental results and comparative performance analysis. Section 5 describes the robot path planning simulations and discusses the corresponding results. Finally, Section 6 concludes the paper and outlines future research directions.

Although the classical BBO algorithm has demonstrated good global search ability, it still suffers from several limitations, including loss of population diversity during the evolutionary process and the risk of premature convergence.

## 2. Biogeography-Based Optimization (BBO)

Biogeography-based optimization (BBO) is a population-based evolutionary optimization algorithm inspired by the theory of biogeography, which studies the geographical distribution of biological species over space and time. In biogeography, the suitability of a habitat determines its ability to support species populations. Habitats with high suitability tend to have abundant species and export them to less suitable habitats, whereas habitats with low suitability are more likely to accept incoming species.

In BBO, each candidate solution is regarded as a habitat, and its quality is measured by the habitat suitability index (HSI), which corresponds to the fitness value of the solution. The variables of a solution are treated as suitability index variables (SIVs), analogous to species in a habitat. The optimization process is mainly driven by two evolutionary operators: migration and mutation.

### 2.1. Migration Mechanism

Migration is the primary information-sharing mechanism in BBO. It models the process by which features of high-quality solutions are probabilistically shared with low-quality solutions. For a population of size *N*, all habitats are ranked according to their HSI values. Habitats with higher HSI values are assigned higher emigration rates and lower immigration rates, while habitats with lower HSI values have higher immigration rates and lower emigration rates.

The immigration rate $\lambda_{i}$ and emigration rate $\mu_{i}$ of the ith habitat are commonly defined as:

(1) $$\;\lambda_{i}=I\left(1-\frac{i}{S_{max}}\right)$$

(2) $$\;\mu_{i}=E\frac{i}{S_{max}}$$

where *I* and *E* denote the maximum immigration and emigration rates, respectively, and *i* is the rank index after sorting habitats by fitness. $S_{max}$ denotes the maximum number of species.

During the migration process, each habitat probabilistically decides whether to modify its SIV based on its immigration rate. If migration occurs, a source habitat is selected according to emigration probabilities and one or more SIVs of the current habitat are replaced by the corresponding SIVs from the selected source habitat. This variable-level information exchange enables inferior solutions to learn partial structures from superior ones while preserving population diversity.

### 2.2. Mutation Operator

Habitat HSI may undergo mutations due to random catastrophic events. Therefore, the BBO algorithm employs a mutation operator to randomly perturb habitats, simulating such mutations to prevent premature convergence and maintain population diversity. The mutation operation uses species population probabilities to determine mutation rates. The mutation rate m(S) is calculated as follows:

(3) $$\;m\left(S\right)=m_{max}\left(\frac{1-P_{s}}{P_{max}}\right)$$

(4) $${\dot{P}}_{s}=\{\begin{array}{c} \begin{array}{cc} -\left(\lambda_{s}+\mu_{s}\right)P_{s}+\mu_{s+1}P_{s+1}, & \;S=0 \end{array}\;\\ \begin{array}{cc} -\left(\lambda_{s}+\mu_{s}\right)P_{s}+\lambda_{s-1}P_{s-1}+\mu_{s+1}P_{s+1}, & 1\leq S\leq S_{max}-1 \end{array}\\ \begin{array}{cc} -\left(\lambda_{s}+\mu_{s}\right)P_{s}+\lambda_{s-1}P_{s-1}, & \;S= \end{array}S_{max}\; \end{array}$$

where S represents the current species count, $m_{max}$ denotes the set maximum mutation rate, $P_{s}$ indicates the probability when the species count is S, $P_{max}$ signifies the maximum value of species probability.

From the formula, it can be deduced that the mutation rate is inversely proportional to the species abundance probability. Species abundance probability is jointly determined by species abundance, immigration rate, and emigration rate. When species abundance is too low or too high, the corresponding species abundance probability is low, resulting in a relatively high mutation rate. Moderate species abundance corresponds to a higher species abundance probability, leading to a lower mutation rate.

### 2.3. Elitism

To ensure that high-quality solutions are not lost during evolution, an elitism strategy is usually adopted. A certain number of elite habitats with the best HSI values are preserved and directly carried over to the next generation.

## 3. Dynamic Multi-Niche Biogeography-Based Optimization (DMBBO)

**Figure 1:**
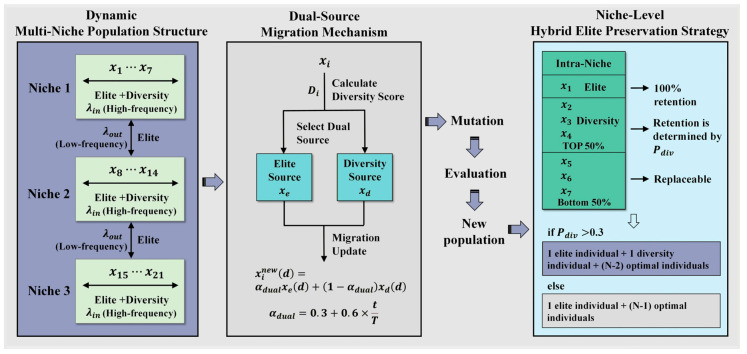
Schematic diagram of DMBBO principle.

To improve the exploration capability and population diversity of the standard biogeography-based optimization, we propose a dynamic multi-niche BBO (DMBBO) algorithm. DMBBO introduces three tightly coupled improvement strategies: a dynamic multi-niche population structure, a dual-source migration mechanism, and a niche-level hybrid elite preservation strategy. A schematic diagram illustrating the overall principle of DMBBO is shown in Figure 1.

### 3.1. Dynamic Multi-Niche Population Structure

Standard biogeography-based optimization (BBO) evolves the population in a fully global manner, which often leads to rapid information diffusion and premature convergence, especially when dealing with complex, multimodal optimization problems. Once the global elite dominates the migration process, population diversity decreases sharply and the search may stagnate in local optima.

To alleviate this issue, a dynamic multi-niche population structure is introduced in DMBBO. By dividing the population into multiple semi-independent niches, the algorithm is able to maintain parallel search behaviors in different regions of the solution space, thereby enhancing diversity preservation and reducing the risk of premature convergence.

Let the population size be N, which is divided into K niches, each containing $n_{s}=N/K$ individuals.

The population can be expressed as:

(5) $$\;\begin{array}{cc} R=\overset{K}{\underset{k=1}{⋃}}R^{\left(k\right)}, & \left|R^{\left(k\right)}\right|=n_{s} \end{array}$$

where $R^{\left(k\right)}$ denotes the kth niche.

Within each niche, individuals are sorted in ascending order according to their fitness values.

Let $i_{local}\in \{1,2,\ldots ,n_{s}\}$ denote the rank of an individual within its niche, where $i_{local}=1$ corresponds to the best individual.

Migration operations are mainly performed within niches, while inter-niche migration is activated with a low probability to avoid premature homogenization.

### 3.2. Dual-Source Migration Mechanism

In the original BBO, migration is mainly driven by elite individuals, which accelerates convergence, but may also cause excessive exploitation and loss of exploration capability. When elite individuals repeatedly act as the sole information donors, structurally similar solutions quickly dominate the population.

To address this limitation, DMBBO proposes a hybrid mechanism combining high-frequency dual-source migration within niches with low-frequency elite migration between niches. Elite individuals within niches and highly diverse individuals jointly participate in information migration, preserving high-quality solution information while introducing structurally diverse search directions. This achieves a better balance between exploitation and exploration. Occasional elite exchange between niches prevents isolation. This hybrid mechanism ensures both exploratory diversity and enhanced convergence efficiency.

#### 3.2.1. Intra-Niche Rank-Based Migration

For the $i_{local}$th individual in a niche, the emigration rate $\mu_{in}\left(i_{local}\right)$ and immigration rate $\lambda_{in}\left(i_{local}\right)$ are defined as:

(6) $$\mu_{in}\left(i_{local}\right)=\frac{i_{local}-1}{n_{s}-1}$$

(7) $$\;\lambda_{in}\left(i_{local}\right)=1-\mu_{in}\left(i_{local}\right)$$

where $\mu_{in}\in [0,1]$ represents the probability that an individual provides information and $\lambda_{in}\in [0,1]$ represents the probability that an individual accepts information.

#### 3.2.2. Composite Diversity Evaluation

To identify diversity-driven donors, both spatial and fitness diversity are considered.

Composite diversity $D_{i}$ is then constructed as:

(8) $$D_{i}=\omega_{s}\left(t\right)D_{i}^{s}+\omega_{f}\left(t\right)D_{i}^{f}$$

(9) $$D_{i}^{s}=\frac{1}{n_{s}-1}\sum_{j\neq i}\left\|x_{i}-x_{j}\right\|$$

(10) $$D_{i}^{f}=\left|f\left(x_{i}\right)-\frac{1}{n_{s}}\sum_{j=1}^{n_{s}}f{(x}_{j})\right|$$

(11) $$\;\omega_{s}\left(t\right)=0.8\times \left(1-\frac{t}{T}\right)+0.2$$

(12) $$\;\omega_{f}\left(t\right)=1-\omega_{s}\left(t\right)$$

where $x_{i}$ and $x_{j}$ denote the positions of the ith and jth individuals, respectively, t denotes the current iteration count, T denotes the maximum number of function evaluations, $D_{i}^{s}$ and $D_{i}^{f}$ denote spatial diversity and fitness diversity, respectively, while $\omega_{s}$ and $\omega_{f}$ denote their respective weights, $f\left(x_{i}\right)$ is the fitness value of the ith individual.

The individual with the maximum $D_{i}$ is selected as the diversity donor $x_{d}$.

#### 3.2.3. Dual-Source Migration Update

Let $x_{e}$ denote the elite individual (rank 1) in the niche and $x_{d}$ denote the selected diversity donor. The dual-source migration update for dimension d is defined as:

(13) $$\;\begin{array}{cc} x_{i}^{new}\left(d\right)=\alpha_{dual}x_{e}\left(d\right)+\left(1-\alpha_{dual}\right)x_{d}\left(d\right), & if\;rand\leq \lambda_{in}\left(i_{local}\right) \end{array}$$

(14) $$\alpha_{dual}=0.3+0.6\times \frac{t}{T}$$

where $x_{i}^{new}$ is the new position of $x_{i}$; $\alpha_{dual}$ is the adaptive mixing coefficient.

This strategy enables a smooth transition from exploration to exploitation during evolution.

#### 3.2.4. Low-Frequency Inter-Niche Migration

To enable controlled global information exchange, inter-niche migration is activated with a fixed low probability $\lambda_{out}$. When activated, the elite individual of a randomly selected niche is used to update the current individual.

### 3.3. Niche-Level Hybrid Elite Preservation Strategy

Although elite preservation can improve convergence stability, global elite retention in BBO may further accelerate population homogenization, particularly in multi-niche environments. Protecting only the best individuals may suppress potentially promising, but structurally distinct solutions.

Therefore, DMBBO adopts a niche-level hybrid elite preservation strategy to balance solution quality and structural diversity during evolution, thereby enhancing the algorithm’s overall robustness. Specifically, for each niche, elite individuals are always retained, while the decision to retain diversity individuals is determined by $P_{div}$:

(15) $$P_{div}=\max\left(\begin{array}{cc} t_{dp}, & 1-{\left(\frac{t}{T}\right)}^{2} \end{array}\right)$$

where $t_{dp}$ denotes the diversity preservation threshold. When $P_{div}>t_{dp},$ enable diversity retention by selecting the individual farthest from the elite from the top 50% of individuals (excluding elite individuals). Otherwise, retain only the elite individual.

Therefore, when $P_{div}>t_{dp}$, the final population retains 1 elite individual, 1 diversity individual, and N−2 optimal individuals selected from all remaining individuals. When $P_{div}=t_{dp}$, the final population retains 1 elite individual and N-1 optimal individuals selected from all remaining individuals.

### 3.4. Pseudocode and Flowchart for DMBBO

**Figure 2:**
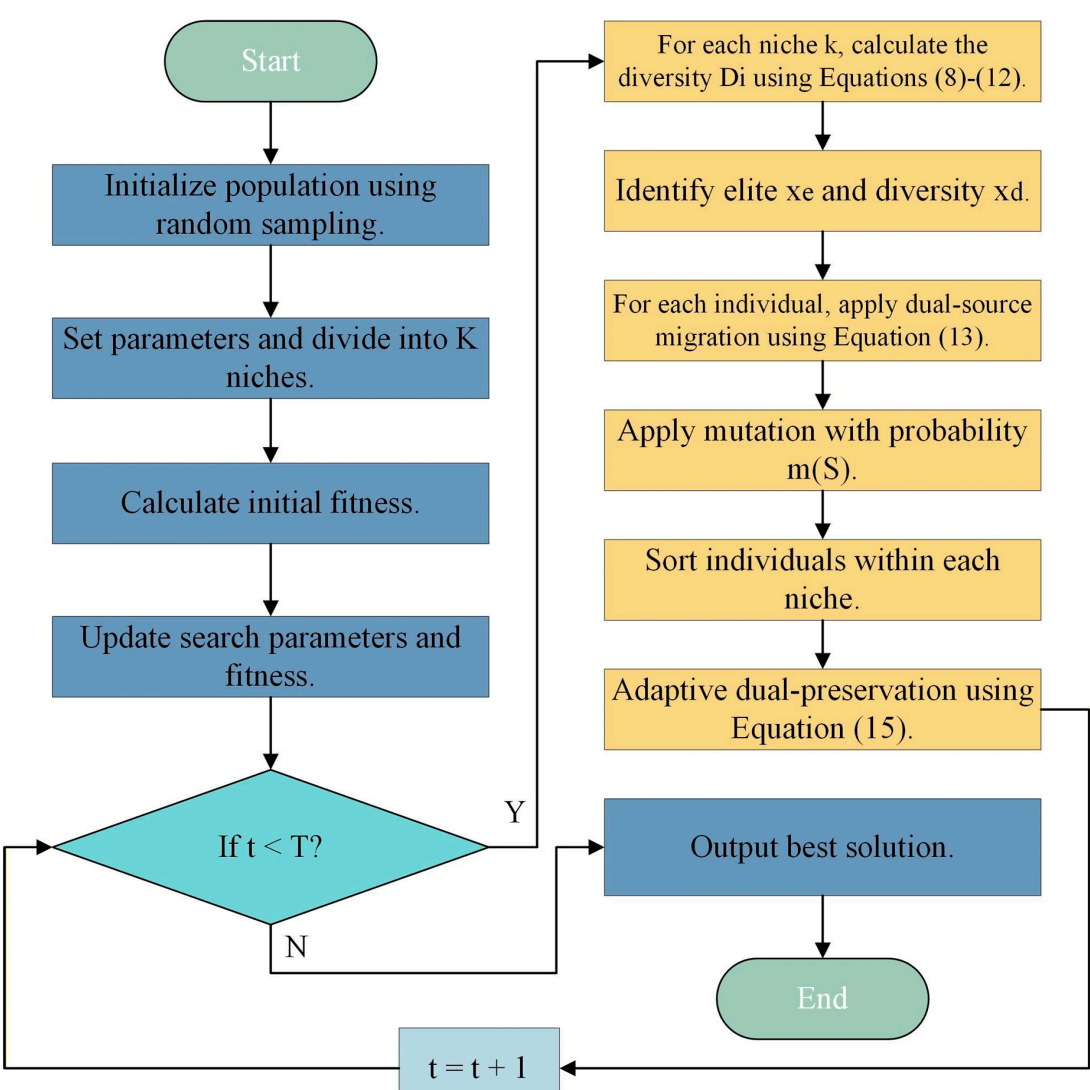
DMBBO flowchart.

The program flowchart of DMBBO, which integrates dynamic multi-niche evolution, dual-source migration, and hybrid elite preservation, is shown in Figure 2. The time complexity analysis is provided in Appendix A.1, and the pseudocode is as follows (Algorithm 1).
**Algorithm 1.** Pseudocode for DMBBOInput: Population size N, maximum evaluations ${FES}_{max}$, bounds lb & ub, dimension dim, objective function objfunOutput: Best solution $X_{best}$, best fitness ${Best}_{FF}$1: Initialize population position using random sampling and evaluate fitness.2: Set parameters: number of niches K = 3, The number of individuals within each niche $n_{s}\;=\;floor(N/K)$, immigration rate $\lambda_{in}$, inter-niche migration rate $\lambda_{out}=0.1$, diversity preservation threshold $t_{dp}=0.3,$ mutation rate m(S)=0.1.3: while t≤ T do4: for each niche k = 1 to K do5: Get current niche population and fitness.6: for each individual i in niche k do7: Calculate $D_{i}^{s}$, $D_{i}^{f}$ $\omega_{s}$ and $\omega_{f}$ using Equations (9)–(12).8: Calculate composite diversity $D_{i}$ using Equation (8).9: end for10: Identify elite $x_{e}$ and diversity donor $x_{d}$.11: for each individual i in niche k do12: Calculate $\lambda_{in}\left(i\right)$ and $\alpha_{dual}$ using Equations (7) and (14).13: for each dimension d do14: if $rand\;\leq \;\lambda_{in}\left(i\right)$ then15: Apply dual-source migration using Equation (13).16: end if17: if $rand\;\leq \;\lambda_{out}$ then18: Set inter-niche elite migration λ__out_ = 0.1.19: end if20: if rand ≤ m(S) then21: Randomly reset SIV.22: end if23: end for24: end for25: end for26: Sort individuals within each niche.27: Calculate $P_{div}$ using Equation (15).28: Preserve elite and diversity individuals.29: end while30: Return $X_{best},$ ${Best}_{FF}$.

## 4. Parameter Sensitivity Analysis

To evaluate the influence of key parameters on the performance of the proposed DMBBO algorithm, a parameter sensitivity analysis is conducted in this section. The proposed algorithm introduces several parameters related to the multi-niche population structure and diversity preservation mechanism, including the niche quantity K, the inter-niche migration rate $\lambda_{out}$, and the diversity preservation threshold $t_{dp}$ used in the population diversity indicator $P_{div}$. Since these parameters may affect the balance between exploration and exploitation, it is necessary to analyze their impact on optimization performance.

In the experiments, the CEC2022 benchmark suite is employed to evaluate the algorithm under different parameter settings. Detailed descriptions of the benchmark functions can be found in Table A1 of Appendix A.2. For each parameter, a range of candidate values is tested while keeping the other parameters fixed. Specifically, the niche quantity K is set to {2, 3, 4, 5, 6}, the inter-niche migration rate $\lambda_{out}$ is selected from {0.05, 0.10, 0.15, 0.20, 0.25}, and the diversity preservation threshold $t_{dp}$ is chosen from {0.1, 0.2, 0.3, 0.4, 0.5}. Each algorithm configuration is independently executed 30 times on each test function to obtain the best value, mean value, and variance of the optimization results.

**Figure 3:**
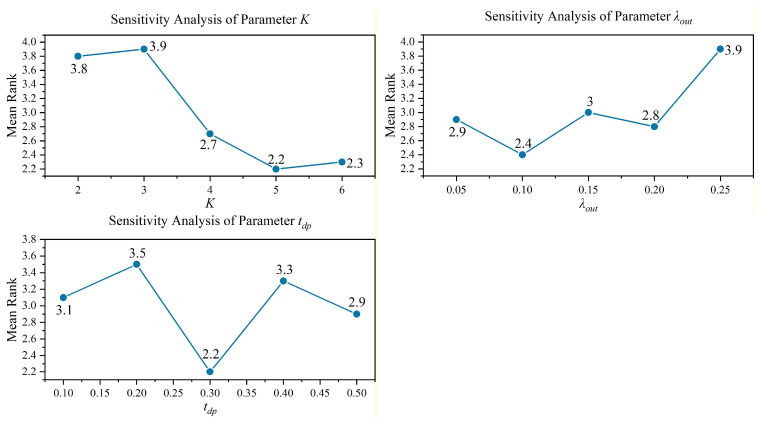
Friedman rankings for parameter sensitivity analysis on CEC2022.

To further evaluate the overall performance under different parameter settings, the Friedman ranking test is employed. The Friedman ranking results are illustrated in Figure 3 To improve the readability of the manuscript, detailed experimental results are provided in Table A4, Table A5 and Table A6 of Appendix A.2.

## 5. Performance Evaluation and Analysis of DMBBO

To comprehensively evaluate the performance of the proposed DMBBO algorithm, three widely used benchmark suites, namely CEC2022, CEC2020, and CEC2019, are employed. Detailed descriptions of CEC2020 and CEC2019 are provided in Table A2 and Table A3 in Appendix A.2.

The experimental study consists of three main parts:

- Ablation experiments conducted on the CEC2022 benchmark to investigate the effectiveness of each proposed strategy.
- Convergence behavior analysis of DMBBO, including the investigation of exploration–exploitation dynamics, search history distribution, trajectories of representative search agents, and convergence curves.
- Comparative performance evaluation, where DMBBO is compared with 23 state-of-the-art algorithms, including classic algorithms, newly proposed algorithms, mature variants, and the original BBO. For each benchmark suite, 12 representative algorithms are selected from the 23 competitors for comparison. Statistical significance of the results is further assessed using the Friedman test and the Wilcoxon rank-sum test.

### 5.1. Experimental Configuration

The compared algorithms and their corresponding parameter settings are summarized in Table 1. The parameter values of all comparative algorithms are adopted from their original publications. Although some algorithms may benefit from problem-specific parameter tuning, using the standard parameter settings reported in the literature is a common practice in metaheuristic optimization studies and helps ensure a fair and reproducible comparison environment.

To ensure a fair comparison, all algorithms are executed under identical experimental conditions. The population size is fixed at 100 for all algorithms. For convergence analysis, the maximum number of iterations is set to 500, while for the remaining experiments, the maximum number of function evaluations is set to 1000× Dim. All algorithms were implemented under the same computational conditions and executed with the same stopping criteria.

To enhance the reliability and reproducibility of the experimental results, each algorithm is independently executed 30 times on each benchmark function. The best, mean, and standard deviation of the obtained solutions are reported. The standard deviation is used to evaluate algorithm stability. Friedman rankings and Wilcoxon rank-sum test results are also presented. All experiments are implemented using MATLAB R2021b.

### 5.2. Ablation Study

To validate the effectiveness of the proposed improvement strategies, two algorithm variants were designed for ablation analysis:

- BBO_D: Incorporates a dual-source migration mechanism based on the original BBO.
- BBO_DM: Further introduces a dynamic multi-niche population structure based on BBO_D.

These variants were compared against the original BBO and the DMBBO algorithm, which simultaneously integrates all three improvement strategies, on a 20-dimensional test function from the CEC2022 benchmark suite. Experimental results include optimal values, mean values, variance, and Friedman rankings to systematically evaluate each improvement strategy’s contribution to algorithmic performance. Relevant experimental results are summarized in Table 2. The optimal result in each row of the table is highlighted in bold and underlined.

As shown in Table 2, all improved algorithm variants achieved overall rankings superior to the original BBO, indicating that the introduced improvement strategies enhanced algorithm performance to varying degrees. Compared to BBO_D, BBO_DM exhibits a slight performance decline. However, when the three strategies synergistically form DMBBO, this algorithm achieves optimal results on most test functions and ranks first in the overall Friedman ranking. This outcome demonstrates that the dynamic multi-niche population structure must synergize with the niche-level hybridization elite retention strategy to fully leverage its advantages in maintaining population diversity and enhancing optimization performance.

### 5.3. Convergence Behavior Analysis

Due to the stochastic nature of population-based metaheuristic algorithms, providing a strict mathematical proof of convergence is generally difficult. Therefore, the convergence behavior of the proposed DMBBO algorithm is analyzed empirically through convergence curves, exploration–exploitation analysis, and search trajectory visualization.

To further analyze the convergence characteristics of the proposed DMBBO algorithm, this section investigates its search behavior from multiple perspectives. The analysis focuses on the exploration–exploitation process, search history distribution, trajectories of representative search agents, and the convergence curve of the best individual. These results provide an intuitive understanding of how DMBBO evolves during the optimization process.

The experiments are carried out on six benchmark functions selected from the CEC2022 test suite. Three functions are taken from the Basic Functions group (F2, F3, and F4), and three functions are taken from the Composition Functions group (F9, F11, and F12). These functions have different landscape features and levels of difficulty, so they are suitable for testing the robustness and convergence behavior of the algorithm.

For each selected benchmark function, the algorithm is executed with a fixed population size. The maximum number of iterations is set to 500 to clearly observe the evolutionary process. Four types of convergence-related results are recorded: the exploration–exploitation curves, the search history distribution, the trajectories of five representative search agents in the first dimension, and the convergence curve of the best individual. The assessment of exploration and development capabilities employs a population-level diversity index [53], calculated using the following formulae:

(16) $$Div=\frac{1}{D}\sum_{j=1}^{D}\frac{1}{N}\left(\sum_{i=1}^{N}\left|median\left(X_{j}\right)-X_{i,j}\right|\right)$$

(17) $$Exploration\%=\frac{Div}{{Div}_{max}}$$

(18) $$\;Exploitation\%=\frac{\left|Div-{Div}_{max}\right|}{{Div}_{max}}$$

where ${Div}_{max}$ represents the population dimensional diversity value, $X_{i,j}$ denotes the position of the ith individual in the jth dimension, $median\left(X_{j}\right)$ is the median of all individual positions in the jth dimension.

**Figure 4:**
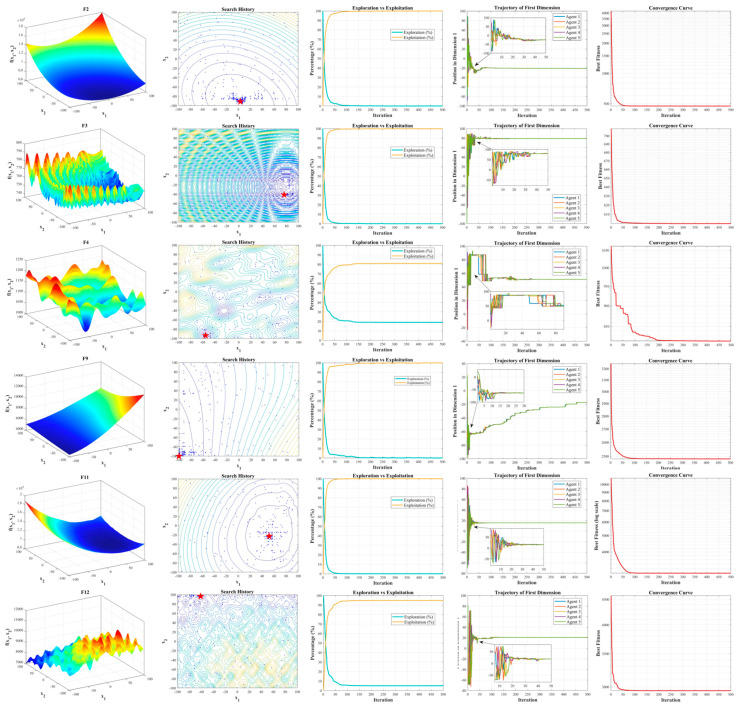
Results of Convergence Behavior Analysis.

The experimental results are shown in Figure 4. The second column in the figure displays the search history of the search agent, with red stars indicating the global optimal solution. At the beginning of the optimization process, search agents are widely scattered across the search space. This indicates sufficient exploration. As the search progresses, an increasing number of agents concentrate around the region near the global optimum. This suggests that promising regions are effectively identified and exploited.

The exploration–exploitation curves in the third column of the figure provides further evidence of this behavior. DMBBO maintains a high exploration level in the early stages of the search. This indicates strong global exploration ability. As the iteration proceeds, the exploration ratio gradually decreases, while the exploitation ratio steadily increases. This trend reflects a smooth transition from exploration to exploitation.

The trajectories of five representative search agents in the first dimension, shown in the fourth column of the figure, describe how individuals move during the optimization process. In the early iterations, the trajectories change widely, which indicates strong global exploration. In later iterations, the trajectories become more stable and gradually focus on certain regions. This shows improved local search ability and more stable convergence.

Finally, the convergence curves of the best individual, shown in the last column of the figure, display a steady improvement in fitness values over iterations. The best solution gradually moves toward the global optimum. This result confirms that DMBBO can effectively guide the population toward high-quality solutions while keeping a stable convergence process.

### 5.4. Test Results and Analysis on CEC2022

Comparative experiments are carried out on 20-dimensional and 10-dimensional problems from the CEC2022 benchmark suite. The results are summarized in Table 3 and Table 4. The tables list the best values, mean values, and Friedman rankings of each algorithm on different test functions. The best results are marked in bold and underlined. In addition, the symbols “+/=/−” show whether DMBBO performs better than, the same as, or worse than the other algorithms on the given metric.

**Figure 5:**
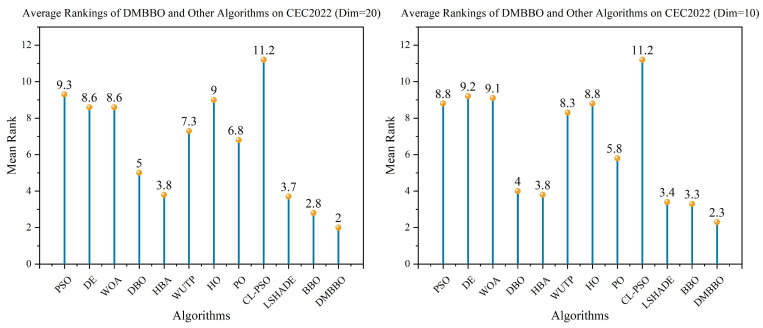
Average Friedman rankings of DMBBO and other algorithms on CEC2022.

**Figure 6:**
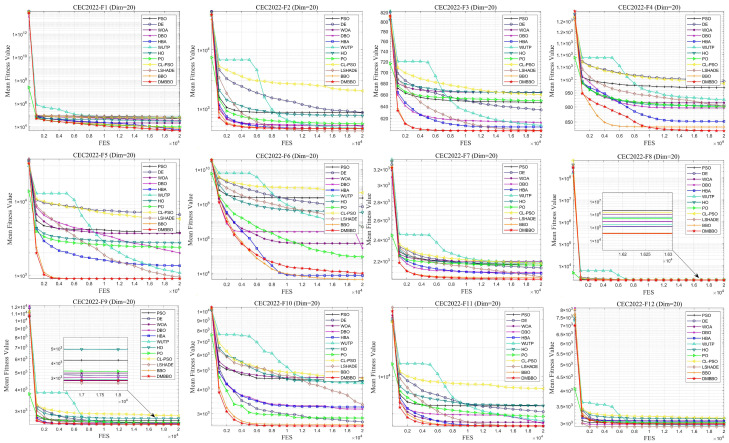
Average iteration curves of DMBBO and other algorithms on CEC2022 (Dim = 20).

**Figure 7:**
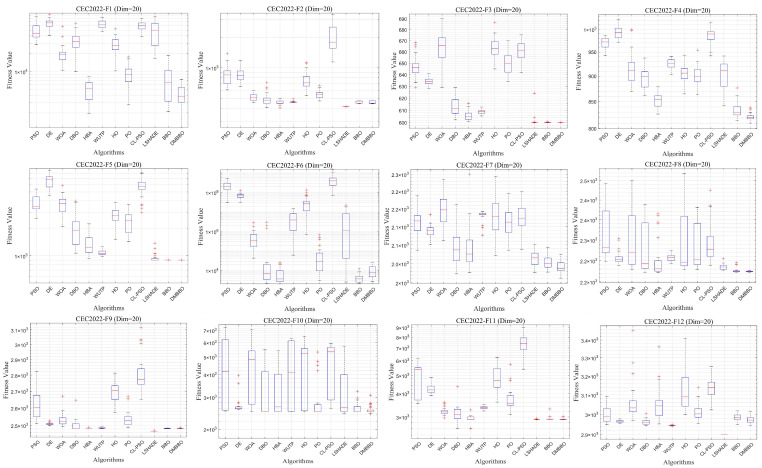
Boxplots of DMBBO and other algorithms on CEC2022 (Dim = 20).

**Figure 8:**
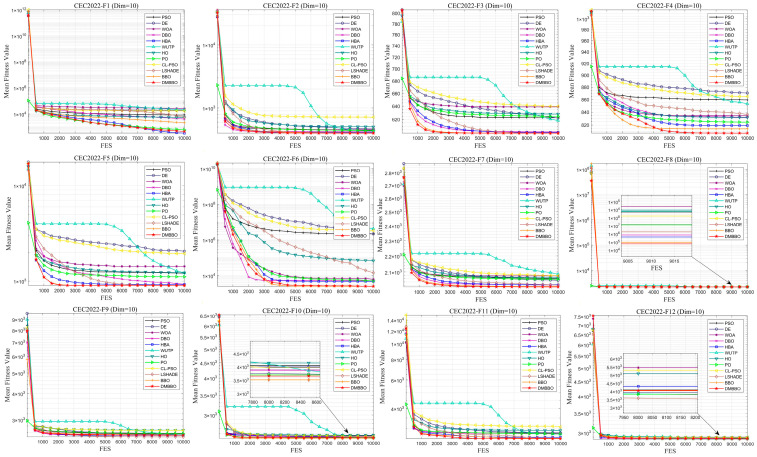
Average iteration curves of DMBBO and other algorithms on CEC2022 (Dim = 10).

**Figure 9:**
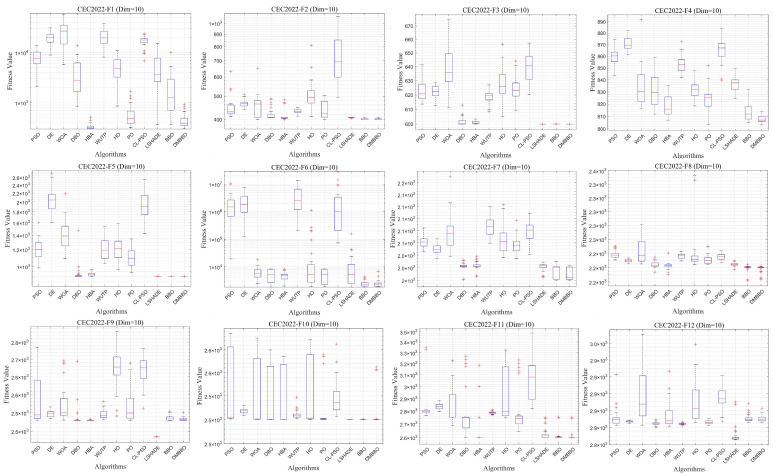
Boxplots of DMBBO and other algorithms on CEC2022 (Dim = 10).

To make it easier to compare the overall performance across different dimensions, Figure 5 shows the average Friedman rankings of all algorithms for each problem dimension. The convergence behavior and stability of the algorithms are further shown using convergence curves and box plots in Figure 6, Figure 7, Figure 8 and Figure 9.

The statistical results show that across 36 evaluation metrics of 12 test problems, DMBBO obtains the largest number of best results among all comparison algorithms. In detail, DMBBO achieves 21 best results on 20-dimensional problems and 17 best results on 10-dimensional problems, and it ranks first in the overall Friedman rankings. This indicates that DMBBO has stable and strong overall performance under different dimensional settings.

From the convergence curves, DMBBO reaches near-optimal solutions within fewer iterations and keeps high accuracy in later iterations. This shows fast convergence speed and high search efficiency. In addition, the Wilcoxon rank-sum test results show that DMBBO performs significantly better than most comparison algorithms. These results further confirm the effectiveness and reliability of DMBBO.

### 5.5. Test Results and Analysis on CEC2020

To further validate the generalization performance and stability of the proposed DMBBO algorithm across different benchmark datasets, comparative experiments were conducted on the CEC2020 test function suite. Experiments were conducted under both 20-dimensional and 10-dimensional settings. Relevant results are summarized in Table 5 and Table 6, including the optimal values, average values, and Friedman rankings for each algorithm. The best results in the tables are highlighted in bold and underlined. The symbols “+/=/−” indicate whether DMBBO outperforms, matches, or underperforms the corresponding comparison algorithm in the respective metric.

**Figure 10:**
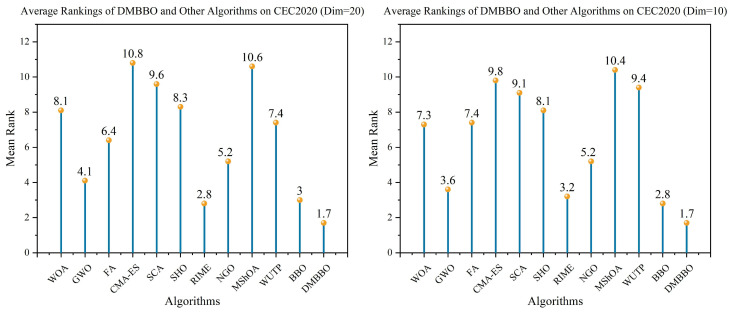
Average Friedman rankings of DMBBO and other algorithms on CEC2020.

**Figure 11:**
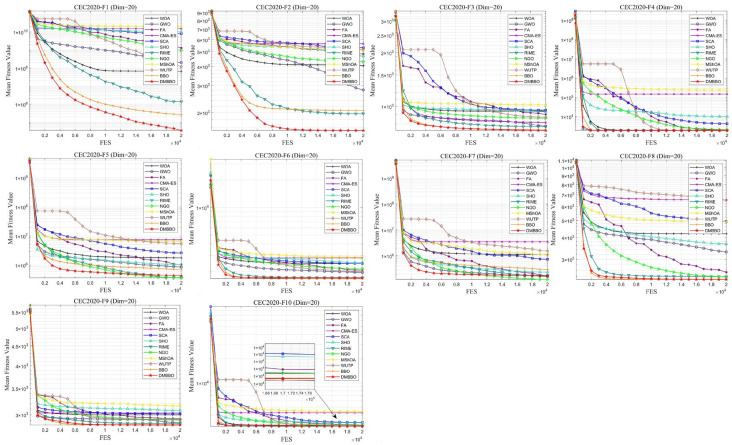
Average iteration curves of DMBBO and other algorithms on CEC2020 (Dim = 20).

**Figure 12:**
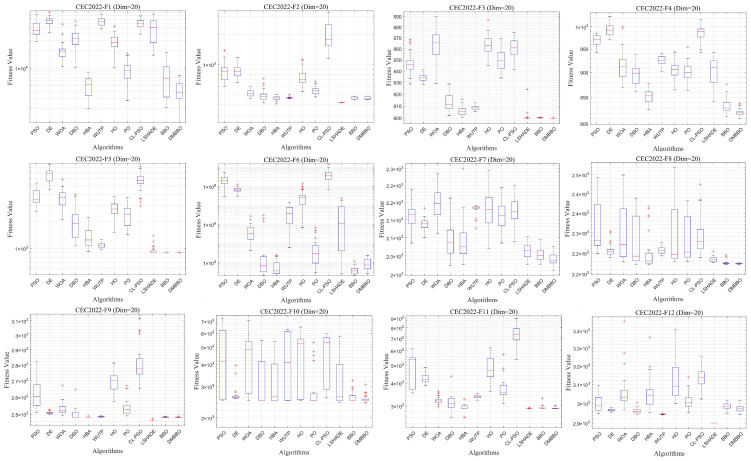
Boxplots of DMBBO and other algorithms on CEC2020 (Dim = 20).

**Figure 13:**
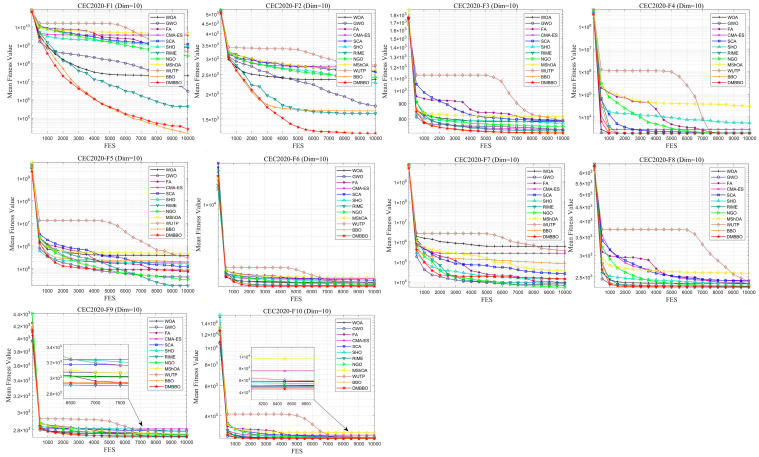
Average iteration curves of DMBBO and other algorithms on CEC2020 (Dim =10).

**Figure 14:**
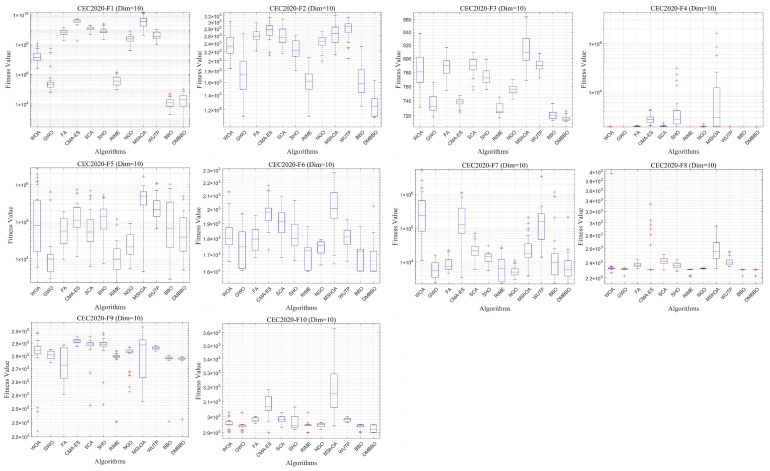
Boxplots of DMBBO and other algorithms on CEC2020 (Dim =10).

To facilitate intuitive comparison of overall performance, Figure 10 presents the average Friedman rank of each algorithm across different dimensions. The convergence behavior and result distribution of each algorithm are illustrated through iterative convergence curves and boxplots, as shown in Figure 11, Figure 12, Figure 13 and Figure 14.

The experimental results demonstrate that for both the 20-dimensional and 10-dimensional problems in CEC2020, DMBBO achieved the best results in 16 out of 30 evaluation metrics and ranked first in the Friedman ranking under both dimensional settings. This shows that DMBBO has strong overall optimization ability across different search space sizes. The results also show that DMBBO can balance global search and local search well on the CEC2020 benchmark suite, which leads to better optimization performance on most test functions.

Analysis of the convergence results shows that DMBBO converges quickly on most test functions and keeps high solution accuracy in the later search stage. This further confirms its search efficiency and stable performance.

### 5.6. Test Results and Analysis on CEC2019

**Figure 15:**
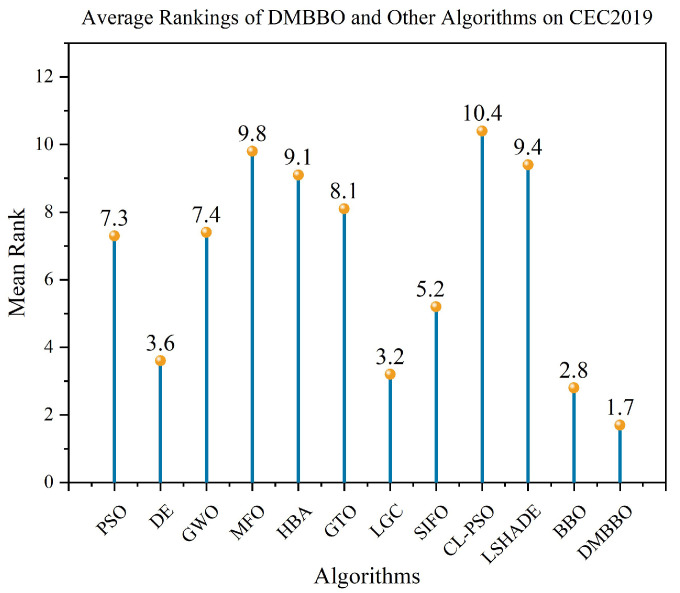
Average Friedman rankings of DMBBO and other algorithms on CEC2019.

**Figure 16:**
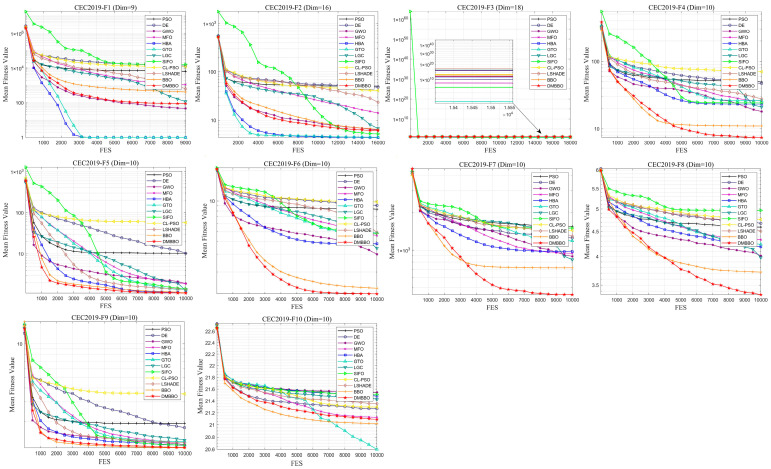
Average iteration curves of DMBBO and other algorithms on CEC2019.

**Figure 17:**
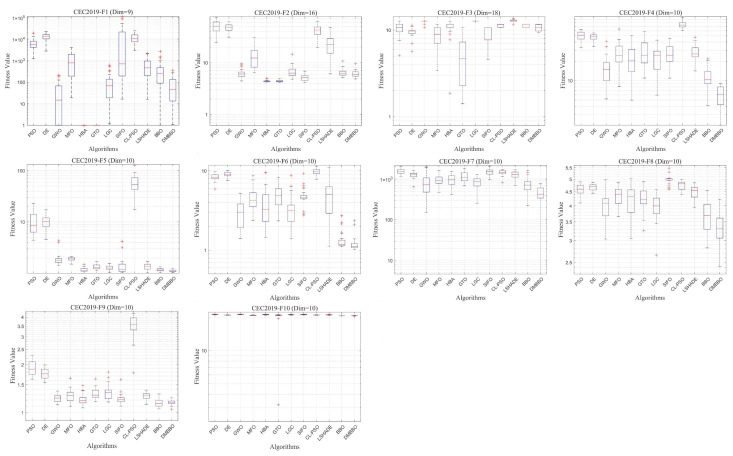
Boxplots of DMBBO and Other Algorithms on CEC2019.

To further test the adaptability of DMBBO on different types of benchmark functions, additional comparison experiments are carried out on the CEC2019 test suite. The experimental settings are the same as those used in the previous sections. The results are shown in Table 7, which includes the best values, mean values, variances, and Friedman rankings of each algorithm on different test functions. The best results are marked in bold and underlined. Figure 15 shows the Friedman ranking results of all algorithms on CEC2019, while Figure 16 and Figure 17 show the convergence curves and box plots of all algorithms.

The statistical results show that among the 30 evaluation metrics in CEC2019, DMBBO achieves the best results on 15 metrics and ranks first in the overall Friedman ranking. This indicates that DMBBO has strong overall performance on this test set. Although some test functions place higher demands on either global search or local search, DMBBO still shows stable and competitive performance in most cases.

Overall, the CEC2019 experimental results show that DMBBO has good robustness and consistent performance across different test suites and problem types. This further confirms the effectiveness of the proposed design in improving search performance and stability.

## 6. Robot Path Planning Simulation and Analysis

### 6.1. Experimental Model

In this study, the mobile robot is modeled as a point and the environment is represented using a grid-based map. Under this model, the path planning problem is treated as a discrete optimization problem, where the robot needs to find a collision-free path from the start point to the goal [54].

The main optimization goal is to minimize the total path length while avoiding obstacles. This helps improve the robot’s movement efficiency and reduce energy use. Therefore, the fitness function is defined as the total Euclidean length of the planned path [55], which is given as follows:

(19) $$\;d=\sum_{k=1}^{n}\sqrt{{\left(x_{k+1}-x_{k}\right)}^{2}+{\left(y_{k+1}-y_{k}\right)}^{2}}$$

where ${(x}_{k},y_{k})$ denotes the coordinates of the kth node, d is the length of the route taken by the vehicle from the point of origin to the final destination, and n is the number of intermediate points along the trip. By minimizing d, the proposed algorithm seeks to find shorter and more efficient paths while meeting the obstacle avoidance constraints of the grid map.

### 6.2. Experimental Configuration for Path Planning

**Figure 18:**
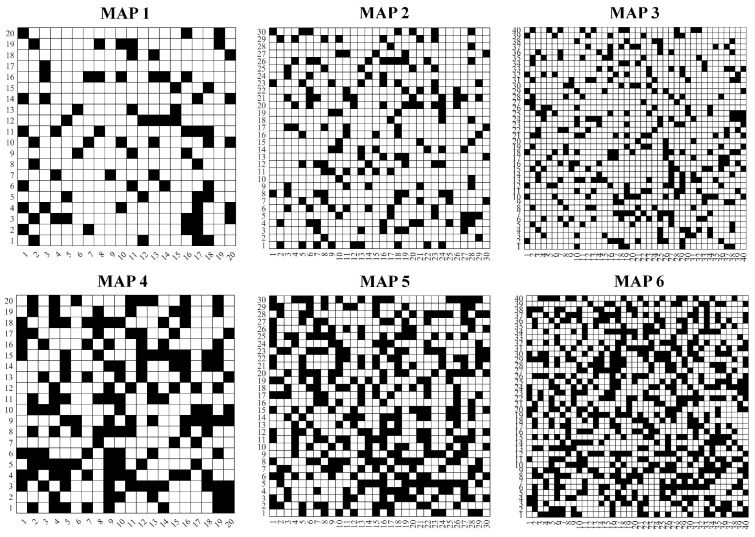
Six simulated map environments.

To fully evaluate the path planning performance of the proposed algorithm under different environment sizes and levels of complexity, three grid map environments are used: 20 × 20, 30 × 30, and 40 × 40. For each map size, obstacle ratios of 20% and 40% were randomly assigned, yielding a total of six experimental environments as shown in Figure 18. Maps 1 to 3 correspond to simple environments with a 20% obstacle ratio, while Maps 4 to 6 represent complex environments with a 40% obstacle ratio.

For algorithm selection, five representative algorithms are selected for comparison, including two classical swarm intelligence algorithms (PSO and GWO), two recently proposed metaheuristic algorithms with strong performance on benchmark problems (GTO and NGO), and the original BBO algorithm. Parameter settings for all comparison algorithms remained consistent with Section 5 to ensure fairness and comparability of experimental results.

The maximum number of function evaluations in each experiment was uniformly set to 20,000. Each algorithm was independently run 30 times under each map environment. Experimental results were statistically analyzed and outputted, including the optimal value, mean, variance, and Friedman ranking of each algorithm’s fitness scores. This comprehensive evaluation assesses the algorithms’ path quality, stability, and overall performance.

### 6.3. Path Planning Results and Analysis in Simple Environments

**Figure 19:**
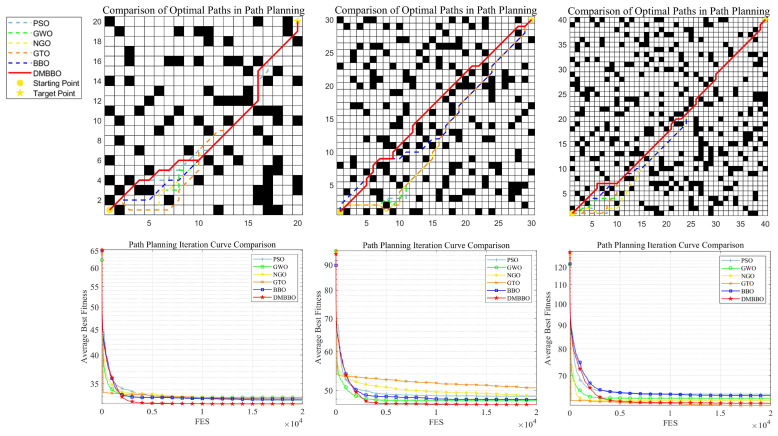
Optimal paths and average iteration curves of DMBBO and other algorithms in a simple environment.

The path planning results in simple environments are summarized in Table 8. Figure 19 shows the planned optimal paths of each algorithm on Maps 1 to 3, as well as their average fitness convergence curves.

The experimental results demonstrate that on Maps 1 and 2, DMBBO consistently achieved the shortest optimal path length and the optimal average path length, exhibiting significant performance advantages. On Map 3, although DMBBO slightly underperformed the GTO algorithm in average path length, it still outperformed the original BBO algorithm and the remaining comparison algorithms, reflecting strong stability and robustness.

Across the three simple map environments with different sizes, DMBBO achieves better results on most evaluation metrics and ranks first in the overall Friedman ranking. This shows that DMBBO can efficiently find high-quality paths in low-complexity environments and maintain stable and strong overall performance across different map sizes.

### 6.4. Path Planning Results and Analysis in Complex Environments

**Figure 20:**
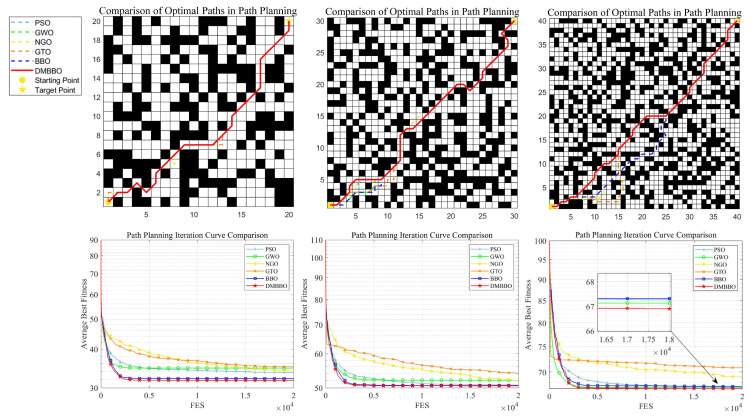
Optimal paths and average iteration curves of DMBBO and other algorithms in a complex environment.

The path planning results in complex environments are summarized in Table 9. Figure 20 shows the optimal paths planned by each algorithm on Maps 4 to 6, along with their average fitness convergence curves.

The results show that in all three complex map environments, DMBBO achieves the shortest best-path length and the best average path length. This indicates clear performance advantages. Even with more obstacles, higher problem dimensions, and more complex path constraints, DMBBO can guide the search effectively, avoid local optima, and continuously generate high-quality feasible paths. Based on all evaluation metrics, DMBBO outperforms all comparison algorithms and ranks first in the Friedman ranking. This further confirms the robustness, stability, and strong path planning ability of the proposed algorithm in highly complex environments.

It should be noted that grid map size may influence task difficulty, as larger maps expand the search space and increase problem dimensionality, potentially adding to optimization complexity. However, DMBBO employs a dynamic multi-niche population structure and adaptive migration strategies to enhance population diversity and global exploration, enabling it to maintain effective search performance even as the search space grows. In practice, algorithm scalability depends more on the efficiency of the underlying search strategy than on map size alone. Given DMBBO’s strong performance on high-dimensional benchmark functions from the CEC test suites, it is expected to maintain robust optimization capability when applied to larger grid maps.

## 7. Conclusions

This paper proposes a new dynamic multi-niche biogeography-based optimization (DMBBO) algorithm to overcome the main weaknesses of the original BBO, including low population diversity, early convergence, and poor balance between global search and local search. To achieve this goal, three improvement strategies are introduced. First, a dynamic multi-niche population structure is used to maintain diversity and support parallel search. Second, a dual-source migration mechanism is designed to improve information sharing while avoiding too much similarity among solutions. Third, a niche-level hybrid elite preservation strategy is applied to keep high-quality solutions and make the convergence process more stable.

The performance of DMBBO is tested on the CEC2022, CEC2020, and CEC2019 benchmark suites under different problem dimensions. Extensive results show that DMBBO performs better than the comparison algorithms in solution accuracy, convergence speed, and overall ranking. Friedman ranking and Wilcoxon rank-sum tests further confirm that these improvements are statistically significant. In addition, ablation experiments verify the individual and combined effects of the proposed strategies, and convergence analysis shows that DMBBO has strong global search ability and stable optimization behavior.

To further test its practical use, DMBBO is applied to robot path planning problems in grid-based environments with different map sizes and obstacle densities. Results from both simple and complex environments show that DMBBO can generate shorter and more stable paths than the comparison algorithms and achieves the best overall performance in most cases. These results indicate that DMBBO remains robust and produces high-quality solutions even in high-dimensional and highly constrained search spaces, which makes it suitable for real-world path planning applications.

Although the DMBBO algorithm demonstrates superior performance in both benchmark function tests and practical applications, several limitations remain. Compared to the original BBO algorithm, the introduction of a dynamic multi-niche structure and hybrid strategies has increased computational complexity to some extent. Furthermore, the algorithm’s performance across different optimization tasks may still be influenced by parameter settings. Consequently, future research will focus on refining its parameter adaptation mechanism and extending DMBBO to more complex large-scale optimization problems and practical engineering applications.

Simultaneously, multiple research directions warrant further exploration. On one hand, extending DMBBO to multi-objective or dynamic optimization scenarios could enhance its applicability in complex robotic tasks, such as multi-robot collaborative path planning and energy-aware navigation. On the other hand, integrating learning-based methods or deploying DMBBO within continuous, high-fidelity robotic simulation environments holds significant research value and application potential.

## Figures and Tables

**Table 1:** Selected comparison algorithms and their parameter settings.

Algorithm	Abbreviation	Parameters Setting	Year	Citation
Classic algorithms				
Particle Swarm Optimization	PSO	$C_{1}=1,C_{2}=1,v_{min}=-10,$ $v_{max}=10,\;\omega =[0.9,0.4]$	1995	[3]
Differential Evolution Algorithm	DE	F=0.5, CR=0.5	1995	[5]
Firefly Algorithm	FA	$\beta_{0}=2,\;\gamma =1,\;\alpha =0.2,$ $\alpha_{d}=0.98$	2008	[6]
Grey Wolf Optimizer	GWO	a=[2, 0]	2014	[7]
Moth-Flame Optimization	MFO	Parameter free	2015	[8]
Whale Optimization Algorithm	WOA	a=[2, 0]	2016	[9]
Covariance Matrix Adaptation—Evolution Strategies	CMA-ES	σ=0.75	2016	[10]
Sine cosine algorithm	SCA	a=2	2016	[11]
Newly proposed algorithms				
Honey Badger Algorithm	HBA	β=6, C=2	2021	[39]
Artificial Gorilla Troops Optimizer	GTO	p=0.03, β=3, ω=0.8	2021	[40]
Dung Beetle Optimizer	DBO	b=0.3	2022	[41]
Sea-Horse Optimizer	SHO	u=0.05, v=0.05, l=0.05	2022	[42]
Northern Goshawk Optimization	NGO	Parameter free	2022	[43]
Rime-ice Optimization	RIME	w=5	2023	[44]
Hippopotamus Optimization Algorithm	HO	Parameter free	2024	[45]
Parrot Optimizer	PO	β=0.5	2024	[46]
Water Uptake and Transport in Plants	WUTP	p=0.5, ρ=1000, η=0.0018, g=9.81, $a=1,\;L_{p}=1\times 10^{-9},$ $D=1\times 10^{-9},\;K=1\times 10^{-9},\;$	2025	[47]
Mantis Shrimp Optimization Algorithm	MShOA	b=1,φ=10	2025	[48]
Logistic-Gauss Circle Optimizer	LGC	$\varepsilon_{min}=0,\;\varepsilon_{max}=0.1$	2025	[49]
Solitary Inchworm Foraging Optimizer	SIFO	α=0.8, m=50, σ=0.5	2025	[50]
Mature variants				
Comprehensive learning particle swarm optimizer	CL-PSO	C=1.2, $F_{max}=7,$ $\omega_{max}=0.9$, $\omega_{min}=0.4$	2006	[51]
Linear Population Size Reduction Success-History Based Adaptive Differential Evolution	LSHADE	F=0.5, CR=0.5	2014	[52]
Original algorithm				
Biogeography-Based Optimization	BBO	I=1, E=1, m(S)=0.1	2008	[16]

**Table 2:** Ablation experiment results.

Function	Index	BBO	BBO_D	BBO_ DM	DMBBO
F1	Best	3.763 × 10^+3^	**3.521 × 10^+2^**	8.063 × 10^+2^	1.568 × 10^+3^
	Mean	1.036 × 10^+4^	**1.727 × 10^+3^**	3.243 × 10^+3^	5.325 × 10^+3^
	Std	5.440 × 10^+3^	**1.609 × 10^+3^**	2.222 × 10^+3^	2.056 × 10^+3^
	Rank	7	1	4	6
F2	Best	4.491 × 10^+2^	4.491 × 10^+2^	**4.453 × 10^+2^**	4.491 × 10^+2^
	Mean	**4.560 × 10^+2^**	4.621 × 10^+2^	4.589 × 10^+2^	4.575 × 10^+2^
	Std	**1.085 × 10^+1^**	1.224 × 10^+1^	1.238 × 10^+1^	1.143 × 10^+1^
	Rank	5	4	2	3
F3	Best	6.001 × 10^+2^	6.001 × 10^+2^	6.001 × 10^+2^	**6.000 × 10^+2^**
	Mean	6.002 × 10^+2^	6.006 × 10^+2^	6.006 × 10^+2^	**6.001 × 10^+2^**
	Std	9.310 × 10^−2^	7.234 × 10^−1^	8.237 × 10^−1^	**5.212 × 10^−2^**
	Rank	3	5	4	1
F4	Best	8.181 × 10^+2^	8.150 × 10^+2^	**8.050 × 10^+2^**	8.162 × 10^+2^
	Mean	8.352 × 10^+2^	8.309 × 10^+2^	**8.219 × 10^+2^**	8.226 × 10^+2^
	Std	1.150 × 10^+1^	1.281 × 10^+1^	9.403 × 10^+0^	**4.054 × 10^+0^**
	Rank	4	3	1	2
F5	Best	9.001 × 10^+2^	9.001 × 10^+2^	**9.000 × 10^+2^**	**9.000 × 10^+2^**
	Mean	9.006 × 10^+2^	9.003 × 10^+2^	9.002 × 10^+2^	**9.001 × 10^+2^**
	Std	5.729 × 10^−1^	3.342 × 10^−1^	2.014 × 10^−1^	**1.223 × 10^−1^**
	Rank	4	3	2	1
F6	Best	1.994 × 10^+3^	**1.879 × 10^+3^**	1.923 × 10^+3^	2.104 × 10^+3^
	Mean	4.884 × 10^+3^	**4.310 × 10^+3^**	4.526 × 10^+3^	7.236 × 10^+3^
	Std	3.406 × 10^+3^	3.441 × 10^+3^	**3.069 × 10^+3^**	3.685 × 10^+3^
	Rank	5	1	3	7
F7	Best	2.025 × 10^+3^	2.027 × 10^+3^	2.026 × 10^+3^	**2.024 × 10^+3^**
	Mean	2.045 × 10^+3^	2.052 × 10^+3^	2.051 × 10^+3^	**2.042 × 10^+3^**
	Std	1.984 × 10^+1^	2.085 × 10^+1^	**1.551 × 10^+1^**	1.944 × 10^+1^
	Rank	2	4	3	1
F8	Best	2.222 × 10^+3^	2.222 × 10^+3^	**2.221 × 10^+3^**	2.222 × 10^+3^
	Mean	2.224 × 10^+3^	2.224 × 10^+3^	2.228 × 10^+3^	**2.224 × 10^+3^**
	Std	1.292 × 10^+0^	1.721 × 10^+0^	2.235 × 10^+1^	**1.099 × 10^+0^**
	Rank	4	3	5	6
F9	Best	**2.481 × 10^+3^**	**2.481 × 10^+3^**	2.482 × 10^+3^	**2.481 × 10^+3^**
	Mean	2.483 × 10^+3^	2.483 × 10^+3^	2.484 × 10^+3^	**2.482 × 10^+3^**
	Std	1.082 × 10^+0^	**9.787 × 10^−1^**	1.902 × 10^+0^	1.160 × 10^+0^
	Rank	3	2	6	1
F10	Best	2.500 × 10^+3^	2.500 × 10^+3^	2.500 × 10^+3^	**2.418 × 10^+3^**
	Mean	2.690 × 10^+3^	2.588 × 10^+3^	2.608 × 10^+3^	**2.563 × 10^+3^**
	Std	3.568 × 10^+2^	2.140 × 10^+2^	2.374 × 10^+2^	**1.007 × 10^+2^**
	Rank	3	1	2	4
F11	Best	2.914 × 10^+3^	2.606 × 10^+3^	2.906 × 10^+3^	**2.602 × 10^+3^**
	Mean	2.925 × 10^+3^	2.917 × 10^+3^	2.924 × 10^+3^	**2.910 × 10^+3^**
	Std	**2.149 × 10^+1^**	6.959 × 10^+1^	3.542 × 10^+1^	7.058 × 10^+1^
	Rank	6	5	4	1
F12	Best	2.947 × 10^+3^	2.983 × 10^+3^	2.977 × 10^+3^	**2.941 × 10^+3^**
	Mean	2.984 × 10^+3^	3.018 × 10^+3^	3.013 × 10^+3^	**2.975 × 10^+3^**
	Std	2.078 × 10^+1^	2.892 × 10^+1^	2.423 × 10^+1^	**1.627 × 10^+1^**
	Rank	2	5	4	1
Mean Rank		4	3.08	3.33	**2.83**
Final Ranking		4	2	3	1

**Table 3:** Comparison of DMBBO with other algorithms on CEC2022 (Dim = 20).

Function	Index	PSO	DE	WOA	DBO	HBA	WUTP	HO	PO	CL-PSO	LSHA-DE	BBO	DM-BBO
F1	Best	2.852 × 10^+4^	4.121 × 10^+4^	1.052 × 10^+4^	1.014 × 10^+4^	1.970 × 10^+3^	4.759 × 10^+4^	1.023 × 10^+4^	2.732 × 10^+3^	3.867 × 10^+4^	1.681 × 10^+4^	2.093 × 10^+3^	**1.136 × 10^+3^**
	Mean	4.884 × 10^+4^	6.551 × 10^+4^	2.012 × 10^+4^	3.301 × 10^+4^	5.079 × 10^+3^	6.328 × 10^+4^	2.890 × 10^+4^	9.232 × 10^+3^	5.907 × 10^+4^	4.858 × 10^+4^	7.094 × 10^+3^	**4.221 × 10^+3^**
	Std	1.490 × 10^+4^	1.081 × 10^+4^	9.043 × 10^+3^	1.211 × 10^+4^	1.915 × 10^+3^	9.874 × 10^+3^	8.801 × 10^+3^	3.540 × 10^+3^	1.106 × 10^+4^	1.983 × 10^+4^	4.399 × 10^+3^	**1.747 × 10^+3^**
	Rank	8	11	5	7	2	12	6	4	10	9	3	1
F2	Best	6.082 × 10^+2^	6.573 × 10^+2^	4.579 × 10^+2^	4.121 × 10^+2^	**4.056 × 10^+2^**	4.552 × 10^+2^	5.338 × 10^+2^	4.738 × 10^+2^	1.144 × 10^+3^	4.167 × 10^+2^	4.451 × 10^+2^	4.449 × 10^+2^
	Mean	8.508 × 10^+2^	8.590 × 10^+2^	5.193 × 10^+2^	4.978 × 10^+2^	4.574 × 10^+2^	4.629 × 10^+2^	7.554 × 10^+2^	5.510 × 10^+2^	2.004 × 10^+3^	**4.180 × 10^+2^**	4.567 × 10^+2^	4.601 × 10^+2^
	Std	1.738 × 10^+2^	1.138 × 10^+2^	4.271 × 10^+1^	6.882 × 10^+1^	1.829 × 10^+1^	7.367 × 10^+0^	1.396 × 10^+2^	4.958 × 10^+1^	6.569 × 10^+2^	**5.396 × 10^−1^**	1.147 × 10^+1^	1.244 × 10^+1^
	Rank	10	11	7	6	2	5	9	8	12	1	3	4
F3	Best	6.293 × 10^+2^	6.285 × 10^+2^	6.295 × 10^+2^	6.023 × 10^+2^	6.009 × 10^+2^	6.055 × 10^+2^	6.450 × 10^+2^	6.341 × 10^+2^	6.417 × 10^+2^	**6.000 × 10^+2^**	6.001 × 10^+2^	**6.000 × 10^+2^**
	Mean	6.469 × 10^+2^	6.343 × 10^+2^	6.645 × 10^+2^	6.132 × 10^+2^	6.063 × 10^+2^	6.088 × 10^+2^	6.637 × 10^+2^	6.499 × 10^+2^	6.602 × 10^+2^	6.010 × 10^+2^	6.003 × 10^+2^	**6.001 × 10^+2^**
	Std	9.390 × 10^+0^	3.030 × 10^+0^	1.400 × 10^+1^	6.957 × 10^+0^	4.148 × 10^+0^	1.761 × 10^+0^	9.115 × 10^+0^	9.258 × 10^+0^	9.579 × 10^+0^	4.472 × 10^+0^	1.490 × 10^−1^	**7.804 × 10^−2^**
	Rank	8	7	11	6	4	5	12	9	10	2	3	1
F4	Best	9.425 × 10^+2^	9.711 × 10^+2^	8.694 × 10^+2^	8.622 × 10^+2^	8.269 × 10^+2^	9.029 × 10^+2^	8.650 × 10^+2^	8.640 × 10^+2^	9.434 × 10^+2^	8.427 × 10^+2^	8.141 × 10^+2^	**8.102 × 10^+2^**
	Mean	9.696 × 10^+2^	9.928 × 10^+2^	9.147 × 10^+2^	8.969 × 10^+2^	8.524 × 10^+2^	9.255 × 10^+2^	9.044 × 10^+2^	9.011 × 10^+2^	9.851 × 10^+2^	9.035 × 10^+2^	8.339 × 10^+2^	**8.218 × 10^+2^**
	Std	1.264 × 10^+1^	1.280 × 10^+1^	3.299 × 10^+1^	2.105 × 10^+1^	1.370 × 10^+1^	9.781 × 10^+0^	1.554 × 10^+1^	1.889 × 10^+1^	1.567 × 10^+1^	2.604 × 10^+1^	1.429 × 10^+1^	**6.916 × 10^+0^**
	Rank	10	12	8	4	3	9	6	5	11	7	2	1
F5	Best	2.572 × 10^+3^	4.563 × 10^+3^	2.085 × 10^+3^	1.073 × 10^+3^	9.254 × 10^+2^	9.763 × 10^+2^	1.516 × 10^+3^	1.424 × 10^+3^	2.990 × 10^+3^	9.000 × 10^+2^	9.002 × 10^+2^	**9.000 × 10^+2^**
	Mean	3.791 × 10^+3^	6.568 × 10^+3^	3.688 × 10^+3^	1.997 × 10^+3^	1.343 × 10^+3^	1.079 × 10^+3^	2.735 × 10^+3^	2.380 × 10^+3^	5.753 × 10^+3^	9.461 × 10^+2^	9.006 × 10^+2^	**9.001 × 10^+2^**
	Std	7.868 × 10^+2^	1.133 × 10^+3^	8.492 × 10^+2^	7.920 × 10^+2^	3.237 × 10^+2^	6.633 × 10^+1^	4.996 × 10^+2^	5.851 × 10^+2^	1.278 × 10^+3^	1.058 × 10^+2^	5.459 × 10^−1^	**2.290 × 10^−1^**
	Rank	9	12	10	6	5	4	8	7	11	3	2	1
F6	Best	3.006 × 10^+7^	3.130 × 10^+7^	4.372 × 10^+4^	2.176 × 10^+3^	**2.052 × 10^+3^**	6.186 × 10^+4^	6.775 × 10^+5^	2.990 × 10^+3^	7.129 × 10^+7^	2.587 × 10^+3^	2.178 × 10^+3^	2.659 × 10^+3^
	Mean	2.207 × 10^+8^	6.950 × 10^+7^	5.224 × 10^+5^	2.763 × 10^+5^	7.349 × 10^+3^	4.999 × 10^+6^	3.229 × 10^+7^	8.786 × 10^+4^	4.489 × 10^+8^	4.678 × 10^+6^	**4.320 × 10^+3^**	9.959 × 10^+3^
	Std	1.417 × 10^+8^	1.773 × 10^+7^	5.737 × 10^+5^	7.286 × 10^+5^	6.666 × 10^+3^	4.304 × 10^+6^	3.045 × 10^+7^	1.552 × 10^+5^	2.945 × 10^+8^	6.730 × 10^+6^	**2.219 × 10^+3^**	6.814 × 10^+3^
	Rank	11	10	6	4	2	8	9	5	12	7	1	3
F7	Best	2.085 × 10^+3^	2.100 × 10^+3^	2.112 × 10^+3^	2.024 × 10^+3^	2.026 × 10^+3^	2.127 × 10^+3^	2.071 × 10^+3^	2.085 × 10^+3^	2.088 × 10^+3^	2.027 × 10^+3^	2.028 × 10^+3^	**2.012 × 10^+3^**
	Mean	2.162 × 10^+3^	2.138 × 10^+3^	2.196 × 10^+3^	2.099 × 10^+3^	2.091 × 10^+3^	2.181 × 10^+3^	2.179 × 10^+3^	2.163 × 10^+3^	2.178 × 10^+3^	2.063 × 10^+3^	2.053 × 10^+3^	**2.042 × 10^+3^**
	Std	3.438 × 10^+1^	1.721 × 10^+1^	4.085 × 10^+1^	5.204 × 10^+1^	5.592 × 10^+1^	**1.406 × 10^+1^**	4.914 × 10^+1^	4.195 × 10^+1^	4.088 × 10^+1^	2.145 × 10^+1^	1.782 × 10^+1^	1.548 × 10^+1^
	Rank	7	6	11	5	4	12	10	8	9	3	2	1
F8	Best	2.248 × 10^+3^	2.239 × 10^+3^	2.229 × 10^+3^	2.222 × 10^+3^	2.222 × 10^+3^	2.243 × 10^+3^	2.229 × 10^+3^	2.230 × 10^+3^	2.239 × 10^+3^	2.228 × 10^+3^	2.223 × 10^+3^	**2.222 × 10^+3^**
	Mean	2.313 × 10^+3^	2.257 × 10^+3^	2.300 × 10^+3^	2.269 × 10^+3^	2.251 × 10^+3^	2.257 × 10^+3^	2.287 × 10^+3^	2.278 × 10^+3^	2.293 × 10^+3^	2.235 × 10^+3^	2.226 × 10^+3^	**2.224 × 10^+3^**
	Std	6.255 × 10^+1^	1.549 × 10^+1^	6.478 × 10^+1^	5.633 × 10^+1^	4.396 × 10^+1^	8.532 × 10^+0^	6.998 × 10^+1^	5.434 × 10^+1^	4.844 × 10^+1^	6.524 × 10^+0^	5.668 × 10^+0^	**1.051 × 10^+0^**
	Rank	12	7	10	5	4	9	6	8	11	3	2	1
F9	Best	2.510 × 10^+3^	2.497 × 10^+3^	2.492 × 10^+3^	2.481 × 10^+3^	2.481 × 10^+3^	2.482 × 10^+3^	2.572 × 10^+3^	2.489 × 10^+3^	2.651 × 10^+3^	**2.465 × 10^+3^**	2.481 × 10^+3^	2.481 × 10^+3^
	Mean	2.616 × 10^+3^	2.508 × 10^+3^	2.530 × 10^+3^	2.498 × 10^+3^	2.481 × 10^+3^	2.486 × 10^+3^	2.690 × 10^+3^	2.539 × 10^+3^	2.798 × 10^+3^	**2.466 × 10^+3^**	2.483 × 10^+3^	2.482 × 10^+3^
	Std	8.080 × 10^+1^	6.451 × 10^+0^	3.443 × 10^+1^	3.212 × 10^+1^	1.488 × 10^+0^	2.675 × 10^+0^	5.758 × 10^+1^	4.769 × 10^+1^	1.152 × 10^+2^	1.494 × 10^+0^	1.412 × 10^+0^	**1.092 × 10^+0^**
	Rank	10	7	8	5	2	6	11	9	12	1	4	3
F10	Best	2.516 × 10^+3^	2.562 × 10^+3^	2.501 × 10^+3^	2.501 × 10^+3^	2.501 × 10^+3^	2.501 × 10^+3^	2.503 × 10^+3^	2.501 × 10^+3^	2.603 × 10^+3^	2.445 × 10^+3^	2.500 × 10^+3^	**2.426 × 10^+3^**
	Mean	4.420 × 10^+3^	2.694 × 10^+3^	4.560 × 10^+3^	3.161 × 10^+3^	3.240 × 10^+3^	4.294 × 10^+3^	4.368 × 10^+3^	2.828 × 10^+3^	4.532 × 10^+3^	3.333 × 10^+3^	2.605 × 10^+3^	**2.559 × 10^+3^**
	Std	1.917 × 10^+3^	2.999 × 10^+2^	1.439 × 10^+3^	9.352 × 10^+2^	9.285 × 10^+2^	1.821 × 10^+3^	1.514 × 10^+3^	7.625 × 10^+2^	1.304 × 10^+3^	1.103 × 10^+3^	1.814 × 10^+2^	**1.356 × 10^+2^**
	Rank	10	7	11	4	5	8	9	3	12	6	2	1
F11	Best	3.530 × 10^+3^	3.890 × 10^+3^	2.963 × 10^+3^	**2.600 × 10^+3^**	2.601 × 10^+3^	3.228 × 10^+3^	3.603 × 10^+3^	3.082 × 10^+3^	5.397 × 10^+3^	2.900 × 10^+3^	2.915 × 10^+3^	2.901 × 10^+3^
	Mean	4.887 × 10^+3^	4.237 × 10^+3^	3.224 × 10^+3^	3.128 × 10^+3^	2.919 × 10^+3^	3.358 × 10^+3^	4.905 × 10^+3^	3.751 × 10^+3^	7.439 × 10^+3^	**2.904 × 10^+3^**	2.944 × 10^+3^	2.923 × 10^+3^
	Std	9.579 × 10^+2^	2.466 × 10^+2^	1.465 × 10^+2^	2.926 × 10^+2^	1.158 × 10^+2^	7.308 × 10^+1^	7.293 × 10^+2^	5.210 × 10^+2^	9.180 × 10^+2^	**1.100 × 10^+1^**	7.486 × 10^+1^	4.064 × 10^+1^
	Rank	10	9	6	5	3	7	11	8	12	1	4	2
F12	Best	2.948 × 10^+3^	2.954 × 10^+3^	2.969 × 10^+3^	2.943 × 10^+3^	2.952 × 10^+3^	2.941 × 10^+3^	2.998 × 10^+3^	2.953 × 10^+3^	3.023 × 10^+3^	**2.900 × 10^+3^**	2.948 × 10^+3^	2.945 × 10^+3^
	Mean	3.000 × 10^+3^	2.965 × 10^+3^	3.062 × 10^+3^	2.962 × 10^+3^	3.051 × 10^+3^	2.945 × 10^+3^	3.121 × 10^+3^	3.010 × 10^+3^	3.137 × 10^+3^	**2.900 × 10^+3^**	2.983 × 10^+3^	2.974 × 10^+3^
	Std	4.229 × 10^+1^	5.446 × 10^+0^	9.935 × 10^+1^	1.464 × 10^+1^	8.877 × 10^+1^	1.330 × 10^+0^	1.041 × 10^+2^	3.995 × 10^+1^	6.330 × 10^+1^	**1.288 × 10^−4^**	1.794 × 10^+1^	1.638 × 10^+1^
	Rank	7	4	10	3	9	2	11	8	12	1	6	5
Mean Rank		9.3	8.6	8.6	5	3.8	7.3	9	6.8	11.2	3.7	2.8	**2**
+/=/−		12/0/0	11/0/1	12/0/0	11/0/1	9/0/3	11/0/1	12/0/0	11/1/0	12/0/0	8/0/4	8/3/1	~

**Table 4:** Comparison of DMBBO with other algorithms on CEC2022 (Dim = 10).

Function	Index	PSO	DE	WOA	DBO	HBA	WUTP	HO	PO	CL-PSO	LSHA-DE	BBO	DM-BBO
F1	Best	2.128 × 10^+3^	8.921 × 10^+3^	5.700 × 10^+3^	8.489 × 10^+2^	**3.009 × 10^+2^**	8.074 × 10^+3^	8.628 × 10^+2^	3.219 × 10^+2^	6.795 × 10^+3^	3.729 × 10^+2^	3.708 × 10^+2^	3.098 × 10^+2^
	Mean	8.290 × 10^+3^	1.960 × 10^+4^	2.577 × 10^+4^	3.998 × 10^+3^	**3.369 × 10^+2^**	2.069 × 10^+4^	5.411 × 10^+3^	5.957 × 10^+2^	1.716 × 10^+4^	5.484 × 10^+3^	2.070 × 10^+3^	4.632 × 10^+2^
	Std	2.971 × 10^+3^	5.606 × 10^+3^	1.325 × 10^+4^	3.143 × 10^+3^	**4.952 × 10^+1^**	6.856 × 10^+3^	2.629 × 10^+3^	3.387 × 10^+2^	3.978 × 10^+3^	4.194 × 10^+3^	2.061 × 10^+3^	1.685 × 10^+2^
	Rank	8	10	12	5	1	11	7	3	9	6	4	2
F2	Best	4.127 × 10^+2^	4.405 × 10^+2^	4.009 × 10^+2^	4.076 × 10^+2^	4.000 × 10^+2^	4.136 × 10^+2^	4.126 × 10^+2^	4.008 × 10^+2^	4.947 × 10^+2^	4.053 × 10^+2^	4.000 × 10^+2^	**4.000 × 10^+2^**
	Mean	4.496 × 10^+2^	4.670 × 10^+2^	4.552 × 10^+2^	4.225 × 10^+2^	4.130 × 10^+2^	4.337 × 10^+2^	5.041 × 10^+2^	4.414 × 10^+2^	7.192 × 10^+2^	4.073 × 10^+2^	4.024 × 10^+2^	**4.017 × 10^+2^**
	Std	4.508 × 10^+1^	1.567 × 10^+1^	5.206 × 10^+1^	2.507 × 10^+1^	2.103 × 10^+1^	8.356 × 10^+0^	7.872 × 10^+1^	3.491 × 10^+1^	1.532 × 10^+2^	**7.104 × 10^−1^**	2.702 × 10^+0^	2.265 × 10^+0^
	Rank	9	10	8	5	4	6	11	7	12	3	2	1
F3	Best	6.136 × 10^+2^	6.125 × 10^+2^	6.115 × 10^+2^	6.000 × 10^+2^	6.001 × 10^+2^	6.086 × 10^+2^	6.052 × 10^+2^	6.095 × 10^+2^	6.202 × 10^+2^	6.000 × 10^+2^	6.000 × 10^+2^	**6.000 × 10^+2^**
	Mean	6.226 × 10^+2^	6.223 × 10^+2^	6.390 × 10^+2^	6.018 × 10^+2^	6.011 × 10^+2^	6.183 × 10^+2^	6.282 × 10^+2^	6.247 × 10^+2^	6.400 × 10^+2^	6.000 × 10^+2^	6.000 × 10^+2^	**6.000 × 10^+2^**
	Std	6.769 × 10^+0^	4.234 × 10^+0^	1.399 × 10^+1^	2.814 × 10^+0^	9.438 × 10^−1^	4.878 × 10^+0^	1.162 × 10^+1^	8.592 × 10^+0^	9.962 × 10^+0^	**7.750 × 10^−3^**	2.962 × 10^−2^	1.195 × 10^−2^
	Rank	7	8	11	4	5	6	10	9	12	1	3	2
F4	Best	8.438 × 10^+2^	8.616 × 10^+2^	8.161 × 10^+2^	8.119 × 10^+2^	8.070 × 10^+2^	8.422 × 10^+2^	8.185 × 10^+2^	8.036 × 10^+2^	8.398 × 10^+2^	8.247 × 10^+2^	8.050 × 10^+2^	**8.032 × 10^+2^**
	Mean	8.594 × 10^+2^	8.707 × 10^+2^	8.346 × 10^+2^	8.322 × 10^+2^	8.192 × 10^+2^	8.530 × 10^+2^	8.317 × 10^+2^	8.244 × 10^+2^	8.651 × 10^+2^	8.365 × 10^+2^	8.142 × 10^+2^	**8.078 × 10^+2^**
	Std	7.023 × 10^+0^	5.854 × 10^+0^	1.593 × 10^+1^	1.334 × 10^+1^	8.382 × 10^+0^	7.073 × 10^+0^	7.770 × 10^+0^	1.023 × 10^+1^	9.999 × 10^+0^	5.542 × 10^+0^	7.832 × 10^+0^	**2.662 × 10^+0^**
	Rank	10	12	7	5	3	9	6	4	11	8	2	1
F5	Best	9.855 × 10^+2^	1.607 × 10^+3^	1.089 × 10^+3^	9.000 × 10^+2^	9.001 × 10^+2^	1.033 × 10^+3^	9.683 × 10^+2^	9.394 × 10^+2^	1.425 × 10^+3^	9.000 × 10^+2^	9.000 × 10^+2^	**9.000 × 10^+2^**
	Mean	1.212 × 10^+3^	2.055 × 10^+3^	1.425 × 10^+3^	9.286 × 10^+2^	9.208 × 10^+2^	1.220 × 10^+3^	1.223 × 10^+3^	1.112 × 10^+3^	1.939 × 10^+3^	9.002 × 10^+2^	9.000 × 10^+2^	**9.000 × 10^+2^**
	Std	1.377 × 10^+2^	2.691 × 10^+2^	2.456 × 10^+2^	1.050 × 10^+2^	2.026 × 10^+1^	1.364 × 10^+2^	1.634 × 10^+2^	1.200 × 10^+2^	2.715 × 10^+2^	5.936 × 10^−1^	5.556 × 10^−2^	**4.214 × 10^−2^**
	Rank	7	12	10	4	5	9	8	6	11	2	3	1
F6	Best	1.933 × 10^+4^	1.218 × 10^+5^	2.441 × 10^+3^	1.845 × 10^+3^	2.004 × 10^+3^	2.150 × 10^+5^	1.933 × 10^+3^	1.903 × 10^+3^	7.350 × 10^+4^	1.830 × 10^+3^	**1.824 × 10^+3^**	1.851 × 10^+3^
	Mean	2.184 × 10^+6^	2.187 × 10^+6^	6.248 × 10^+3^	5.201 × 10^+3^	4.699 × 10^+3^	4.207 × 10^+6^	6.864 × 10^+4^	5.013 × 10^+3^	3.279 × 10^+6^	1.438 × 10^+4^	2.522 × 10^+3^	**2.508 × 10^+3^**
	Std	2.490 × 10^+6^	1.759 × 10^+6^	3.132 × 10^+3^	2.515 × 10^+3^	1.744 × 10^+3^	3.645 × 10^+6^	2.122 × 10^+5^	2.445 × 10^+3^	5.642 × 10^+6^	2.877 × 10^+4^	**7.632 × 10^+2^**	9.805 × 10^+2^
	Rank	9	11	8	3	5	12	7	4	10	6	2	1
F7	Best	2.046 × 10^+3^	2.034 × 10^+3^	2.038 × 10^+3^	2.001 × 10^+3^	2.007 × 10^+3^	2.060 × 10^+3^	2.036 × 10^+3^	2.034 × 10^+3^	2.041 × 10^+3^	2.004 × 10^+3^	2.000 × 10^+3^	**2.000 × 10^+3^**
	Mean	2.062 × 10^+3^	2.050 × 10^+3^	2.077 × 10^+3^	2.022 × 10^+3^	2.024 × 10^+3^	2.087 × 10^+3^	2.065 × 10^+3^	2.057 × 10^+3^	2.078 × 10^+3^	2.021 × 10^+3^	2.013 × 10^+3^	**2.011 × 10^+3^**
	Std	9.750 × 10^+0^	7.149 × 10^+0^	2.802 × 10^+1^	**6.370 × 10^+0^**	6.599 × 10^+0^	1.543 × 10^+1^	2.170 × 10^+1^	1.429 × 10^+1^	1.775 × 10^+1^	6.675 × 10^+0^	1.043 × 10^+1^	1.013 × 10^+1^
	Rank	8	6	10	3	4	12	9	7	11	5	2	1
F8	Best	2.231 × 10^+3^	2.226 × 10^+3^	2.225 × 10^+3^	2.214 × 10^+3^	2.210 × 10^+3^	2.229 × 10^+3^	2.225 × 10^+3^	2.224 × 10^+3^	2.227 × 10^+3^	2.218 × 10^+3^	**2.202 × 10^+3^**	2.204 × 10^+3^
	Mean	2.238 × 10^+3^	2.230 × 10^+3^	2.242 × 10^+3^	2.224 × 10^+3^	2.223 × 10^+3^	2.237 × 10^+3^	2.240 × 10^+3^	2.231 × 10^+3^	2.236 × 10^+3^	2.225 × 10^+3^	2.220 × 10^+3^	**2.218 × 10^+3^**
	Std	5.290 × 10^+0^	**2.014 × 10^+0^**	1.639 × 10^+1^	3.870 × 10^+0^	4.805 × 10^+0^	3.647 × 10^+0^	3.027 × 10^+1^	5.421 × 10^+0^	4.311 × 10^+0^	2.268 × 10^+0^	4.760 × 10^+0^	5.060 × 10^+0^
	Rank	12	7	9	4	3	11	8	6	10	5	2	1
F9	Best	2.530 × 10^+3^	2.534 × 10^+3^	2.530 × 10^+3^	2.529 × 10^+3^	2.529 × 10^+3^	2.531 × 10^+3^	2.541 × 10^+3^	2.530 × 10^+3^	2.563 × 10^+3^	**2.486 × 10^+3^**	2.529 × 10^+3^	2.529 × 10^+3^
	Mean	2.582 × 10^+3^	2.547 × 10^+3^	2.571 × 10^+3^	2.538 × 10^+3^	2.530 × 10^+3^	2.546 × 10^+3^	2.673 × 10^+3^	2.575 × 10^+3^	2.670 × 10^+3^	**2.486 × 10^+3^**	2.536 × 10^+3^	2.533 × 10^+3^
	Std	6.479 × 10^+1^	8.615 × 10^+0^	4.951 × 10^+1^	3.156 × 10^+1^	1.091 × 10^+0^	1.140 × 10^+1^	5.153 × 10^+1^	5.138 × 10^+1^	3.951 × 10^+1^	**1.844 × 10^−1^**	6.234 × 10^+0^	4.778 × 10^+0^
	Rank	10	7	9	3	2	6	11	8	12	1	5	4
F10	Best	2.501 × 10^+3^	2.508 × 10^+3^	2.501 × 10^+3^	**2.500 × 10^+3^**	**2.500 × 10^+3^**	**2.502 × 10^+3^**	2.501 × 10^+3^	**2.500 × 10^+3^**	2.507 × 10^+3^	**2.500 × 10^+3^**	**2.500 × 10^+3^**	**2.500 × 10^+3^**
	Mean	2.554 × 10^+3^	2.518 × 10^+3^	2.539 × 10^+3^	2.535 × 10^+3^	2.549 × 10^+3^	2.510 × 10^+3^	2.563 × 10^+3^	2.519 × 10^+3^	2.546 × 10^+3^	**2.501 × 10^+3^**	2.501 × 10^+3^	2.515 × 10^+3^
	Std	7.722 × 10^+1^	5.681 × 10^+0^	6.467 × 10^+1^	5.758 × 10^+1^	6.036 × 10^+1^	9.354 × 10^+0^	7.195 × 10^+1^	4.556 × 10^+1^	3.454 × 10^+1^	**1.009 × 10^−1^**	1.727 × 10^−1^	3.696 × 10^+1^
	Rank	10	11	7	4	6	8	9	5	12	2	1	3
F11	Best	2.765 × 10^+3^	2.796 × 10^+3^	2.687 × 10^+3^	2.600 × 10^+3^	2.600 × 10^+3^	2.769 × 10^+3^	2.748 × 10^+3^	2.642 × 10^+3^	2.818 × 10^+3^	2.600 × 10^+3^	2.601 × 10^+3^	**2.600 × 10^+3^**
	Mean	2.829 × 10^+3^	2.835 × 10^+3^	2.820 × 10^+3^	2.782 × 10^+3^	2.664 × 10^+3^	2.786 × 10^+3^	2.932 × 10^+3^	2.796 × 10^+3^	3.080 × 10^+3^	2.626 × 10^+3^	2.618 × 10^+3^	**2.611 × 10^+3^**
	Std	1.395 × 10^+2^	2.495 × 10^+1^	1.335 × 10^+2^	1.930 × 10^+2^	1.574 × 10^+2^	**9.166 × 10^+0^**	2.168 × 10^+2^	1.749 × 10^+2^	1.879 × 10^+2^	5.012 × 10^+1^	4.517 × 10^+1^	3.672 × 10^+1^
	Rank	9	11	7	5	2	8	10	6	12	1	4	3
F12	Best	2.863 × 10^+3^	2.865 × 10^+3^	2.863 × 10^+3^	2.860 × 10^+3^	2.861 × 10^+3^	2.862 × 10^+3^	2.866 × 10^+3^	2.862 × 10^+3^	2.867 × 10^+3^	**2.846 × 10^+3^**	2.866 × 10^+3^	2.865 × 10^+3^
	Mean	2.871 × 10^+3^	2.867 × 10^+3^	2.899 × 10^+3^	2.864 × 10^+3^	2.876 × 10^+3^	2.864 × 10^+3^	2.891 × 10^+3^	2.866 × 10^+3^	2.895 × 10^+3^	**2.851 × 10^+3^**	2.871 × 10^+3^	2.870 × 10^+3^
	Std	1.176 × 10^+1^	9.245 × 10^−1^	3.084 × 10^+1^	1.781 × 10^+0^	1.892 × 10^+1^	**8.448 × 10^−1^**	2.482 × 10^+1^	2.105 × 10^+0^	1.422 × 10^+1^	1.074 × 10^+1^	4.547 × 10^+0^	3.958 × 10^+0^
	Rank	7	5	11	3	6	2	10	4	12	1	9	8
Mean Rank		8.8	9.2	9.1	4	3.8	8.3	8.8	5.8	11.2	3.4	3.3	**2.3**
+/=/−		11/1/0	11/0/1	12/0/0	11/0/1	8/2/2	10/0/2	12/0/0	10/1/1	12/0/0	8/2/2	7/5/0	~

**Table 5:** Comparison of DMBBO with other algorithms on CEC2020 (Dim = 20).

Function	Index	WOA	GWO	FA	CMA-ES	SCA	SHO	RIME	NGO	MShOA	WUTP	BBO	DM-BBO
F1	Best	2.060 × 10^+3^	1.695 × 10^+3^	1.902 × 10^+3^	2.443 × 10^+3^	2.051 × 10^+3^	2.033 × 10^+3^	1.610 × 10^+3^	1.894 × 10^+3^	2.190 × 10^+3^	1.794 × 10^+3^	1.603 × 10^+3^	**1.602 × 10^+3^**
	Mean	2.443 × 10^+3^	1.898 × 10^+3^	2.033 × 10^+3^	2.861 × 10^+3^	2.487 × 10^+3^	2.503 × 10^+3^	1.763 × 10^+3^	2.197 × 10^+3^	2.970 × 10^+3^	2.004 × 10^+3^	1.719 × 10^+3^	**1.701 × 10^+3^**
	Std	2.479 × 10^+2^	1.547 × 10^+2^	9.715 × 10^+1^	2.136 × 10^+2^	1.845 × 10^+2^	2.604 × 10^+2^	9.283 × 10^+1^	1.517 × 10^+2^	3.760 × 10^+2^	1.014 × 10^+2^	7.044 × 10^+1^	**9.145 × 10^+1^**
	Rank	9	4	6	12	10	8	3	7	11	5	2	1
F2	Best	2.318 × 10^+3^	2.313 × 10^+3^	2.457 × 10^+3^	3.280 × 10^+3^	2.806 × 10^+3^	2.693 × 10^+3^	2.306 × 10^+3^	2.347 × 10^+3^	3.008 × 10^+3^	2.710 × 10^+3^	2.305 × 10^+3^	**2.300 × 10^+3^**
	Mean	4.194 × 10^+3^	3.309 × 10^+3^	2.533 × 10^+3^	6.542 × 10^+3^	4.928 × 10^+3^	3.674 × 10^+3^	2.386 × 10^+3^	2.377 × 10^+3^	4.901 × 10^+3^	6.532 × 10^+3^	2.307 × 10^+3^	**2.303 × 10^+3^**
	Std	1.920 × 10^+3^	1.455 × 10^+3^	5.423 × 10^+1^	**9.145 × 10^+2^**	1.951 × 10^+3^	1.165 × 10^+3^	4.144 × 10^+2^	1.899 × 10^+1^	1.030 × 10^+3^	9.770 × 10^+2^	9.547 × 10^−1^	6.262 × 10^+0^
	Rank	7	6	5	11	10	8	3	4	9	12	2	1
F3	Best	8.316 × 10^+2^	7.481 × 10^+2^	8.397 × 10^+2^	7.931 × 10^+2^	9.006 × 10^+2^	8.634 × 10^+2^	7.450 × 10^+2^	8.212 × 10^+2^	9.059 × 10^+2^	8.462 × 10^+2^	7.298 × 10^+2^	**7.268 × 10^+2^**
	Mean	9.422 × 10^+2^	7.745 × 10^+2^	9.025 × 10^+2^	8.135 × 10^+2^	9.541 × 10^+2^	9.350 × 10^+2^	7.696 × 10^+2^	8.600 × 10^+2^	1.020 × 10^+3^	8.700 × 10^+2^	7.392 × 10^+2^	**7.336 × 10^+2^**
	Std	4.993 × 10^+1^	1.904 × 10^+1^	1.866 × 10^+1^	7.195 × 10^+0^	2.694 × 10^+1^	2.928 × 10^+1^	1.570 × 10^+1^	1.803 × 10^+1^	4.530 × 10^+1^	9.860 × 10^+0^	4.940 × 10^+0^	**3.218 × 10^+0^**
	Rank	10	4	8	5	11	9	3	6	12	7	2	1
F4	Best	1.915 × 10^+3^	1.904 × 10^+3^	1.932 × 10^+3^	1.836 × 10^+4^	2.321 × 10^+3^	3.401 × 10^+3^	1.902 × 10^+3^	1.928 × 10^+3^	4.633 × 10^+3^	1.911 × 10^+3^	**1.902 × 10^+3^**	1.903 × 10^+3^
	Mean	1.945 × 10^+3^	1.909 × 10^+3^	1.984 × 10^+3^	1.468 × 10^+5^	4.296 × 10^+3^	1.008 × 10^+4^	1.906 × 10^+3^	2.085 × 10^+3^	2.423 × 10^+5^	1.914 × 10^+3^	1.904 × 10^+3^	**1.904 × 10^+3^**
	Std	2.446 × 10^+1^	2.993 × 10^+0^	5.233 × 10^+1^	9.993 × 10^+4^	1.575 × 10^+3^	7.573 × 10^+3^	1.955 × 10^+0^	1.895 × 10^+2^	2.499 × 10^+5^	1.545 × 10^+0^	1.101 × 10^+0^	**9.645 × 10^−1^**
	Rank	6	4	7	11	9	10	3	8	12	5	1	2
F5	Best	5.958 × 10^+4^	4.849 × 10^+4^	4.898 × 10^+5^	8.972 × 10^+5^	3.085 × 10^+5^	4.057 × 10^+5^	**3.709 × 10^+4^**	1.150 × 10^+5^	5.579 × 10^+5^	5.788 × 10^+5^	4.319 × 10^+4^	1.131 × 10^+5^
	Mean	1.765 × 10^+6^	8.547 × 10^+5^	1.038 × 10^+6^	7.459 × 10^+6^	2.608 × 10^+6^	1.062 × 10^+6^	3.678 × 10^+5^	3.608 × 10^+5^	**6.559 × 10^+6^**	5.344 × 10^+6^	6.979 × 10^+5^	4.346 × 10^+5^
	Std	1.559 × 10^+6^	9.021 × 10^+5^	4.500 × 10^+5^	5.017 × 10^+6^	1.410 × 10^+6^	5.656 × 10^+5^	3.041 × 10^+5^	**1.752 × 10^+5^**	6.479 × 10^+6^	2.389 × 10^+6^	5.105 × 10^+5^	3.299 × 10^+5^
	Rank	8	4	7	12	9	6	1	2	10	11	5	3
F6	Best	2.060 × 10^+3^	1.695 × 10^+3^	1.902 × 10^+3^	2.443 × 10^+3^	2.051 × 10^+3^	2.033 × 10^+3^	1.610 × 10^+3^	1.894 × 10^+3^	2.190 × 10^+3^	1.794 × 10^+3^	1.603 × 10^+3^	**1.602 × 10^+3^**
	Mean	2.443 × 10^+3^	1.898 × 10^+3^	2.033 × 10^+3^	2.861 × 10^+3^	2.487 × 10^+3^	2.503 × 10^+3^	1.763 × 10^+3^	2.197 × 10^+3^	2.970 × 10^+3^	2.004 × 10^+3^	1.719 × 10^+3^	**1.701 × 10^+3^**
	Std	2.479 × 10^+2^	1.547 × 10^+2^	9.715 × 10^+1^	2.136 × 10^+2^	1.845 × 10^+2^	2.604 × 10^+2^	9.283 × 10^+1^	1.517 × 10^+2^	3.760 × 10^+2^	1.014 × 10^+2^	**7.044 × 10^+1^**	9.145 × 10^+1^
	Rank	9	4	6	12	10	8	3	7	11	5	2	1
F7	Best	5.038 × 10^+4^	7.824 × 10^+3^	3.302 × 10^+4^	2.890 × 10^+5^	1.186 × 10^+5^	6.424 × 10^+4^	**5.402 × 10^+3^**	4.859 × 10^+4^	6.503 × 10^+4^	1.977 × 10^+5^	6.608 × 10^+3^	8.258 × 10^+3^
	Mean	1.141 × 10^+6^	1.592 × 10^+5^	1.721 × 10^+5^	3.543 × 10^+6^	7.418 × 10^+5^	2.368 × 10^+5^	1.757 × 10^+5^	**1.227 × 10^+5^**	1.117 × 10^+6^	1.552 × 10^+6^	2.930 × 10^+5^	1.660 × 10^+5^
	Std	9.535 × 10^+5^	1.694 × 10^+5^	1.078 × 10^+5^	3.002 × 10^+6^	7.153 × 10^+5^	2.085 × 10^+5^	1.764 × 10^+5^	**6.071 × 10^+4^**	9.312 × 10^+5^	8.809 × 10^+5^	2.457 × 10^+5^	1.746 × 10^+5^
	Rank	10	1	5	12	8	6	4	2	9	11	7	3
F8	Best	2.318 × 10^+3^	2.313 × 10^+3^	2.457 × 10^+3^	3.280 × 10^+3^	2.806 × 10^+3^	2.693 × 10^+3^	2.306 × 10^+3^	2.347 × 10^+3^	3.008 × 10^+3^	2.710 × 10^+3^	2.305 × 10^+3^	**2.300 × 10^+3^**
	Mean	4.194 × 10^+3^	3.309 × 10^+3^	2.533 × 10^+3^	6.542 × 10^+3^	4.928 × 10^+3^	3.674 × 10^+3^	2.386 × 10^+3^	2.377 × 10^+3^	4.901 × 10^+3^	6.532 × 10^+3^	2.307 × 10^+3^	**2.303 × 10^+3^**
	Std	1.920 × 10^+3^	1.455 × 10^+3^	5.423 × 10^+1^	9.145 × 10^+2^	1.951 × 10^+3^	1.165 × 10^+3^	4.144 × 10^+2^	1.899 × 10^+1^	1.030 × 10^+3^	9.770 × 10^+2^	**9.547 × 10^−1^**	6.262 × 10^+0^
	Rank	7	6	5	11	10	8	3	4	9	12	2	1
F9	Best	2.877 × 10^+3^	2.821 × 10^+3^	2.896 × 10^+3^	2.985 × 10^+3^	2.981 × 10^+3^	3.007 × 10^+3^	2.828 × 10^+3^	2.876 × 10^+3^	3.006 × 10^+3^	2.888 × 10^+3^	**2.806 × 10^+3^**	**2.806 × 10^+3^**
	Mean	2.995 × 10^+3^	2.865 × 10^+3^	2.930 × 10^+3^	3.029 × 10^+3^	3.013 × 10^+3^	3.073 × 10^+3^	2.854 × 10^+3^	2.918 × 10^+3^	3.155 × 10^+3^	2.911 × 10^+3^	2.836 × 10^+3^	**2.829 × 10^+3^**
	Std	6.114 × 10^+1^	4.010 × 10^+1^	1.396 × 10^+1^	1.680 × 10^+1^	1.702 × 10^+1^	3.591 × 10^+1^	2.021 × 10^+1^	1.678 × 10^+1^	1.036 × 10^+2^	**1.049 × 10^+1^**	1.482 × 10^+1^	1.356 × 10^+1^
	Rank	8	4	7	10	9	11	3	6	12	5	2	1
F10	Best	2.947 × 10^+3^	2.916 × 10^+3^	2.988 × 10^+3^	3.215 × 10^+3^	3.122 × 10^+3^	3.078 × 10^+3^	**2.913 × 10^+3^**	2.986 × 10^+3^	3.180 × 10^+3^	2.918 × 10^+3^	2.914 × 10^+3^	2.914 × 10^+3^
	Mean	3.033 × 10^+3^	2.961 × 10^+3^	3.041 × 10^+3^	4.281 × 10^+3^	3.281 × 10^+3^	3.252 × 10^+3^	2.942 × 10^+3^	3.024 × 10^+3^	4.398 × 10^+3^	**2.926 × 10^+3^**	2.969 × 10^+3^	2.961 × 10^+3^
	Std	4.094 × 10^+1^	3.447 × 10^+1^	3.085 × 10^+1^	5.615 × 10^+2^	1.438 × 10^+2^	1.220 × 10^+2^	3.089 × 10^+1^	1.605 × 10^+1^	1.017 × 10^+3^	**7.364 × 10^+0^**	2.160 × 10^+1^	3.231 × 10^+1^
	Rank	7	4	8	12	10	9	2	6	11	1	5	3
Mean Rank		8.1	4.1	6.4	10.8	9.6	8.3	2.8	5.2	10.6	7.4	3	**1.7**
+/=/−		12/0/0	10/2/0	11/1/0	12/0/0	12/0/0	11/1/0	9/3/0	10/2/0	11/0/1	11/1/0	7/5/0	~

**Table 6:** Comparison of DMBBO with other algorithms on CEC2020 (Dim = 10).

Function	Index	WOA	GWO	FA	CMA-ES	SCA	SHO	RIME	NGO	MShOA	WUTP	BBO	DMBBO
F1	Best	2.494 × 10^+6^	6.212 × 10^+4^	1.798 × 10^+8^	1.727 × 10^+8^	4.734 × 10^+8^	2.222 × 10^+8^	8.771 × 10^+4^	3.770 × 10^+7^	4.255 × 10^+8^	1.018 × 10^+8^	1.919 × 10^+3^	**2.819 × 10^+2^**
	Mean	2.219 × 10^+7^	2.997 × 10^+6^	7.206 × 10^+8^	3.615 × 10^+9^	1.157 × 10^+9^	8.529 × 10^+8^	4.309 × 10^+5^	2.566 × 10^+8^	4.327 × 10^+9^	4.417 × 10^+8^	**1.494 × 10^+4^**	2.527 × 10^+4^
	Std	2.263 × 10^+7^	1.079 × 10^+7^	3.257 × 10^+8^	1.109 × 10^+9^	3.521 × 10^+8^	4.902 × 10^+8^	3.241 × 10^+5^	1.434 × 10^+8^	3.583 × 10^+9^	2.611 × 10^+8^	**1.218 × 10^+4^**	2.357 × 10^+4^
	Rank	5	3	8	12	10	9	4	6	11	7	1	2
F2	Best	1.838 × 10^+3^	1.122 × 10^+3^	2.216 × 10^+3^	2.125 × 10^+3^	2.163 × 10^+3^	1.806 × 10^+3^	1.123 × 10^+3^	1.996 × 10^+3^	2.126 × 10^+3^	2.043 × 10^+3^	1.240 × 10^+3^	**1.107 × 10^+3^**
	Mean	2.359 × 10^+3^	1.742 × 10^+3^	2.597 × 10^+3^	2.744 × 10^+3^	2.577 × 10^+3^	2.268 × 10^+3^	1.597 × 10^+3^	2.425 × 10^+3^	2.629 × 10^+3^	2.780 × 10^+3^	1.647 × 10^+3^	**1.275 × 10^+3^**
	Std	3.240 × 10^+2^	3.775 × 10^+2^	1.689 × 10^+2^	2.334 × 10^+2^	2.487 × 10^+2^	2.379 × 10^+2^	2.317 × 10^+2^	1.649 × 10^+2^	2.468 × 10^+2^	2.456 × 10^+2^	2.893 × 10^+2^	**1.530 × 10^+2^**
	Rank	6	4	10	11	8	5	3	7	9	12	2	1
F3	Best	7.319 × 10^+2^	7.183 × 10^+2^	7.547 × 10^+2^	7.250 × 10^+2^	7.553 × 10^+2^	7.562 × 10^+2^	7.171 × 10^+2^	7.424 × 10^+2^	7.684 × 10^+2^	7.721 × 10^+2^	7.136 × 10^+2^	**7.128 × 10^+2^**
	Mean	7.858 × 10^+2^	7.371 × 10^+2^	7.884 × 10^+2^	7.382 × 10^+2^	7.896 × 10^+2^	7.747 × 10^+2^	7.295 × 10^+2^	7.561 × 10^+2^	8.142 × 10^+2^	7.907 × 10^+2^	7.214 × 10^+2^	**7.164 × 10^+2^**
	Std	2.680 × 10^+1^	1.240 × 10^+1^	1.527 × 10^+1^	5.245 × 10^+0^	1.331 × 10^+1^	1.129 × 10^+1^	7.487 × 10^+0^	6.646 × 10^+0^	2.302 × 10^+1^	8.979 × 10^+0^	6.580 × 10^+0^	**3.441 × 10^+0^**
	Rank	8	4	10	5	9	7	3	6	12	11	2	1
F4	Best	1.903 × 10^+3^	1.901 × 10^+3^	1.908 × 10^+3^	2.055 × 10^+3^	1.912 × 10^+3^	1.914 × 10^+3^	1.901 × 10^+3^	1.902 × 10^+3^	1.907 × 10^+3^	1.904 × 10^+3^	1.900 × 10^+3^	**1.900 × 10^+3^**
	Mean	1.911 × 10^+3^	1.903 × 10^+3^	1.945 × 10^+3^	2.811 × 10^+3^	1.982 × 10^+3^	5.370 × 10^+3^	1.902 × 10^+3^	1.930 × 10^+3^	2.913 × 10^+4^	1.908 × 10^+3^	1.901 × 10^+3^	**1.901 × 10^+3^**
	Std	7.295 × 10^+0^	9.137 × 10^−1^	3.764 × 10^+1^	6.108 × 10^+2^	7.464 × 10^+1^	6.830 × 10^+3^	7.637 × 10^−1^	5.439 × 10^+1^	8.086 × 10^+4^	3.067 × 10^+0^	5.748 × 10^−1^	**4.228 × 10^−1^**
	Rank	6	4	8	12	9	11	3	7	10	5	2	1
F5	Best	5.885 × 10^+3^	2.867 × 10^+3^	9.464 × 10^+3^	1.124 × 10^+4^	6.057 × 10^+3^	7.535 × 10^+3^	**2.287 × 10^+3^**	5.352 × 10^+3^	4.428 × 10^+3^	6.507 × 10^+4^	2.826 × 10^+3^	4.972 × 10^+3^
	Mean	3.912 × 10^+5^	4.234 × 10^+4^	7.231 × 10^+4^	1.930 × 10^+5^	1.223 × 10^+5^	1.671 × 10^+5^	**1.751 × 10^+4^**	3.279 × 10^+4^	4.744 × 10^+5^	3.113 × 10^+5^	1.976 × 10^+5^	8.497 × 10^+4^
	Std	6.682 × 10^+5^	1.302 × 10^+5^	5.115 × 10^+4^	1.955 × 10^+5^	1.663 × 10^+5^	1.399 × 10^+5^	**2.395 × 10^+4^**	2.613 × 10^+4^	3.554 × 10^+5^	2.632 × 10^+5^	2.665 × 10^+5^	1.117 × 10^+5^
	Rank	8	1	5	10	6	9	2	3	11	12	7	4
F6	Best	1.655 × 10^+3^	1.604 × 10^+3^	1.681 × 10^+3^	1.729 × 10^+3^	1.680 × 10^+3^	1.664 × 10^+3^	1.601 × 10^+3^	1.636 × 10^+3^	1.646 × 10^+3^	1.656 × 10^+3^	1.601 × 10^+3^	**1.601 × 10^+3^**
	Mean	1.826 × 10^+3^	1.758 × 10^+3^	1.797 × 10^+3^	1.976 × 10^+3^	1.911 × 10^+3^	1.833 × 10^+3^	1.711 × 10^+3^	1.744 × 10^+3^	2.007 × 10^+3^	1.801 × 10^+3^	1.692 × 10^+3^	**1.667 × 10^+3^**
	Std	9.854 × 10^+1^	1.224 × 10^+2^	7.912 × 10^+1^	9.744 × 10^+1^	8.922 × 10^+1^	1.071 × 10^+2^	9.485 × 10^+1^	**4.306 × 10^+1^**	1.491 × 10^+2^	6.608 × 10^+1^	8.495 × 10^+1^	1.032 × 10^+2^
	Rank	8	5	6	12	10	9	3	4	11	7	2	1
F7	Best	1.104 × 10^+4^	2.494 × 10^+3^	4.603 × 10^+3^	3.517 × 10^+3^	5.910 × 10^+3^	5.530 × 10^+3^	2.339 × 10^+3^	3.064 × 10^+3^	3.820 × 10^+3^	1.384 × 10^+4^	2.277 × 10^+3^	**2.203 × 10^+3^**
	Mean	6.025 × 10^+5^	6.881 × 10^+3^	9.434 × 10^+3^	2.697 × 10^+5^	2.613 × 10^+4^	1.402 × 10^+4^	8.699 × 10^+3^	**5.469 × 10^+3^**	3.655 × 10^+4^	3.654 × 10^+5^	8.429 × 10^+4^	1.438 × 10^+4^
	Std	1.031 × 10^+6^	4.058 × 10^+3^	4.638 × 10^+3^	3.155 × 10^+5^	1.649 × 10^+4^	4.976 × 10^+3^	7.742 × 10^+3^	**2.003 × 10^+3^**	4.399 × 10^+4^	8.294 × 10^+5^	2.581 × 10^+5^	3.710 × 10^+4^
	Rank	12	2	5	10	9	7	3	1	8	11	6	4
F8	Best	2.265 × 10^+3^	2.223 × 10^+3^	2.314 × 10^+3^	2.300 × 10^+3^	2.299 × 10^+3^	2.284 × 10^+3^	**2.212 × 10^+3^**	2.312 × 10^+3^	2.314 × 10^+3^	2.346 × 10^+3^	2.223 × 10^+3^	2.223 × 10^+3^
	Mean	2.372 × 10^+3^	2.309 × 10^+3^	2.369 × 10^+3^	2.430 × 10^+3^	2.418 × 10^+3^	2.361 × 10^+3^	2.295 × 10^+3^	2.319 × 10^+3^	2.579 × 10^+3^	2.408 × 10^+3^	2.300 × 10^+3^	**2.299 × 10^+3^**
	Std	3.014 × 10^+2^	1.809 × 10^+1^	2.727 × 10^+1^	2.803 × 10^+2^	4.627 × 10^+1^	4.128 × 10^+1^	2.957 × 10^+1^	**4.346 × 10^+0^**	1.550 × 10^+2^	5.358 × 10^+1^	1.446 × 10^+1^	1.444 × 10^+1^
	Rank	6	5	9	3	11	8	4	7	12	10	2	1
F9	Best	**2.468 × 10^+3^**	2.722 × 10^+3^	2.601 × 10^+3^	2.787 × 10^+3^	2.561 × 10^+3^	2.564 × 10^+3^	2.501 × 10^+3^	2.611 × 10^+3^	2.576 × 10^+3^	2.767 × 10^+3^	2.503 × 10^+3^	2.511 × 10^+3^
	Mean	2.754 × 10^+3^	2.752 × 10^+3^	**2.718 × 10^+3^**	2.808 × 10^+3^	2.785 × 10^+3^	2.785 × 10^+3^	2.724 × 10^+3^	2.748 × 10^+3^	2.756 × 10^+3^	2.780 × 10^+3^	2.733 × 10^+3^	2.731 × 10^+3^
	Std	8.386 × 10^+1^	1.516 × 10^+1^	6.618 × 10^+1^	8.992 × 10^+0^	4.826 × 10^+1^	5.238 × 10^+1^	7.566 × 10^+1^	4.708 × 10^+1^	8.687 × 10^+1^	**6.135 × 10^+0^**	4.378 × 10^+1^	4.178 × 10^+1^
	Rank	7	5	4	12	11	10	3	6	8	9	2	1
F10	Best	2.901 × 10^+3^	2.901 × 10^+3^	2.956 × 10^+3^	2.898 × 10^+3^	2.933 × 10^+3^	2.917 × 10^+3^	2.898 × 10^+3^	2.920 × 10^+3^	2.941 × 10^+3^	2.961 × 10^+3^	2.899 × 10^+3^	**2.898 × 10^+3^**
	Mean	2.954 × 10^+3^	2.941 × 10^+3^	2.980 × 10^+3^	3.074 × 10^+3^	2.983 × 10^+3^	2.967 × 10^+3^	2.939 × 10^+3^	2.946 × 10^+3^	3.173 × 10^+3^	2.980 × 10^+3^	2.936 × 10^+3^	**2.923 × 10^+3^**
	Std	2.792 × 10^+1^	2.192 × 10^+1^	1.335 × 10^+1^	7.376 × 10^+1^	2.031 × 10^+1^	4.794 × 10^+1^	2.644 × 10^+1^	1.369 × 10^+1^	1.571 × 10^+2^	**1.081 × 10^+1^**	2.043 × 10^+1^	2.366 × 10^+1^
	Rank	7	3	9	11	8	6	4	5	12	10	2	1
Mean Rank		7.3	3.6	7.4	9.8	9.1	8.1	3.2	5.2	10.4	9.4	2.8	**1.7**
+/=/−		11/1/0	9/1/2	9/3/0	11/1/0	11/1/0	11/0/1	9/1/2	9/1/2	11/1/0	12/0/0	6/6/0	~

**Table 7:** Comparison of DMBBO with other algorithms on CEC2019.

Function	Index	PSO	DE	GWO	MFO	HBA	GTO	LGC	SIFO	CL-PSO	LSHA-DE	BBO	DM-BBO
F1	Best	1.226 × 10^+3^	2.790 × 10^+3^	1.000 × 10^+0^	1.898 × 10^+1^	1.000 × 10^+0^	**1.000 × 10^+0^**	1.183 × 10^+0^	1.642 × 10^+1^	3.007 × 10^+3^	1.620 × 10^+1^	1.008 × 10^+0^	1.068 × 10^+0^
	Mean	6.388 × 10^+3^	1.326 × 10^+4^	4.688 × 10^+1^	1.120 × 10^+3^	1.000 × 10^+0^	**1.000 × 10^+0^**	1.181 × 10^+2^	1.635 × 10^+4^	1.183 × 10^+4^	6.303 × 10^+2^	4.165 × 10^+2^	8.853 × 10^+1^
	Std	3.721 × 10^+3^	4.311 × 10^+3^	6.310 × 10^+1^	1.091 × 10^+3^	2.269 × 10^−7^	**0.000 × 10^+0^**	1.550 × 10^+2^	2.778 × 10^+4^	5.513 × 10^+3^	5.749 × 10^+2^	5.689 × 10^+2^	9.605 × 10^+1^
	Rank	10	12	3	8	2	1	5	9	11	7	6	4
F2	Best	2.484 × 10^+1^	3.127 × 10^+1^	4.450 × 10^+0^	6.384 × 10^+0^	4.252 × 10^+0^	4.220 × 10^+0^	4.797 × 10^+0^	**4.121 × 10^+0^**	1.980 × 10^+1^	6.100 × 10^+0^	5.201 × 10^+0^	4.862 × 10^+0^
	Mean	5.084 × 10^+1^	4.841 × 10^+1^	6.324 × 10^+0^	1.416 × 10^+1^	**4.433 × 10^+0^**	4.490 × 10^+0^	6.771 × 10^+0^	5.240 × 10^+0^	4.132 × 10^+1^	2.364 × 10^+1^	6.485 × 10^+0^	6.125 × 10^+0^
	Std	1.375 × 10^+1^	7.473 × 10^+0^	1.271 × 10^+0^	7.043 × 10^+0^	**2.311 × 10^−1^**	2.823 × 10^−1^	1.865 × 10^+0^	7.602 × 10^−1^	1.094 × 10^+1^	1.056 × 10^+1^	1.146 × 10^+0^	9.538 × 10^−1^
	Rank	11	12	5	8	1	2	7	3	10	9	6	4
F3	Best	5.104 × 10^+0^	5.689 × 10^+0^	1.076 × 10^+1^	3.423 × 10^+0^	1.870 × 10^+0^	**1.411 × 10^+0^**	1.271 × 10^+1^	4.609 × 10^+0^	1.066 × 10^+1^	1.171 × 10^+1^	1.071 × 10^+1^	9.423 × 10^+0^
	Mean	1.070 × 10^+1^	9.528 × 10^+0^	1.261 × 10^+1^	8.943 × 10^+0^	1.049 × 10^+1^	**5.027 × 10^+0^**	1.271 × 10^+1^	8.124 × 10^+0^	1.127 × 10^+1^	1.288 × 10^+1^	1.125 × 10^+1^	1.069 × 10^+1^
	Std	1.702 × 10^+0^	1.085 × 10^+0^	3.940 × 10^−1^	2.410 × 10^+0^	2.081 × 10^+0^	2.666 × 10^+0^	**2.245 × 10^−5^**	2.208 × 10^+0^	4.821 × 10^−1^	3.658 × 10^−1^	5.066 × 10^−1^	7.883 × 10^−1^
	Rank	7	3	10	4	6	1	11	2	8	12	9	5
F4	Best	3.239 × 10^+1^	3.437 × 10^+1^	5.192 × 10^+0^	8.142 × 10^+0^	4.980 × 10^+0^	1.099 × 10^+1^	5.977 × 10^+0^	1.098 × 10^+1^	6.038 × 10^+1^	1.419 × 10^+1^	4.131 × 10^+0^	**2.024 × 10^+0^**
	Mean	5.024 × 10^+1^	4.786 × 10^+1^	1.554 × 10^+1^	2.733 × 10^+1^	2.176 × 10^+1^	2.887 × 10^+1^	2.286 × 10^+1^	2.631 × 10^+1^	7.525 × 10^+1^	2.776 × 10^+1^	1.143 × 10^+1^	**6.205 × 10^+0^**
	Std	8.475 × 10^+0^	5.223 × 10^+0^	7.867 × 10^+0^	1.183 × 10^+1^	1.086 × 10^+1^	1.204 × 10^+1^	1.064 × 10^+1^	9.859 × 10^+0^	8.796 × 10^+0^	7.738 × 10^+0^	4.605 × 10^+0^	**2.086 × 10^+0^**
	Rank	11	10	3	6	4	9	5	7	12	8	2	1
F5	Best	4.434 × 10^+0^	4.646 × 10^+0^	1.433 × 10^+0^	1.518 × 10^+0^	**1.007 × 10^+0^**	1.142 × 10^+0^	1.056 × 10^+0^	1.042 × 10^+0^	1.738 × 10^+1^	1.018 × 10^+0^	1.034 × 10^+0^	1.023 × 10^+0^
	Mean	1.033 × 10^+1^	1.035 × 10^+1^	1.922 × 10^+0^	1.915 × 10^+0^	1.177 × 10^+0^	1.360 × 10^+0^	1.307 × 10^+0^	1.431 × 10^+0^	5.714 × 10^+1^	1.366 × 10^+0^	1.172 × 10^+0^	**1.120 × 10^+0^**
	Std	5.067 × 10^+0^	3.137 × 10^+0^	6.443 × 10^−1^	1.428 × 10^−1^	1.159 × 10^−1^	1.674 × 10^−1^	1.173 × 10^−1^	6.609 × 10^−1^	2.368 × 10^+1^	1.945 × 10^−1^	7.014 × 10^−2^	**6.558 × 10^−2^**
	Rank	11	10	8	9	2	7	5	4	12	6	3	1
F6	Best	5.895 × 10^+0^	7.576 × 10^+0^	1.405 × 10^+0^	2.371 × 10^+0^	1.456 × 10^+0^	2.334 × 10^+0^	1.392 × 10^+0^	2.756 × 10^+0^	7.799 × 10^+0^	1.127 × 10^+0^	1.116 × 10^+0^	**1.018 × 10^+0^**
	Mean	8.322 × 10^+0^	9.064 × 10^+0^	2.988 × 10^+0^	4.574 × 10^+0^	3.799 × 10^+0^	4.879 × 10^+0^	3.435 × 10^+0^	4.824 × 10^+0^	9.790 × 10^+0^	4.916 × 10^+0^	1.390 × 10^+0^	**1.218 × 10^+0^**
	Std	8.357 × 10^−1^	6.480 × 10^−1^	1.140 × 10^+0^	1.558 × 10^+0^	2.083 × 10^+0^	1.479 × 10^+0^	1.540 × 10^+0^	1.294 × 10^+0^	9.157 × 10^−1^	2.615 × 10^+0^	4.519 × 10^−1^	**2.915 × 10^−1^**
	Rank	10	11	3	7	5	9	4	8	12	6	2	1
F7	Best	1.163 × 10^+3^	6.419 × 10^+2^	1.520 × 10^+2^	4.563 × 10^+2^	4.164 × 10^+2^	6.757 × 10^+2^	2.498 × 10^+2^	9.602 × 10^+2^	8.108 × 10^+2^	6.953 × 10^+2^	2.195 × 10^+2^	**4.653 × 10^+0^**
	Mean	1.553 × 10^+3^	1.249 × 10^+3^	8.881 × 10^+2^	9.386 × 10^+2^	9.700 × 10^+2^	1.185 × 10^+3^	8.377 × 10^+2^	1.503 × 10^+3^	1.442 × 10^+3^	1.290 × 10^+3^	7.203 × 10^+2^	**4.370 × 10^+2^**
	Std	2.205 × 10^+2^	1.936 × 10^+2^	5.503 × 10^+2^	2.653 × 10^+2^	2.967 × 10^+2^	3.137 × 10^+2^	2.478 × 10^+2^	2.622 × 10^+2^	1.974 × 10^+2^	2.300 × 10^+2^	3.352 × 10^+2^	**1.919 × 10^+2^**
	Rank	12	8	4	5	6	7	3	11	10	9	2	1
F8	Best	4.101 × 10^+0^	4.450 × 10^+0^	3.040 × 10^+0^	3.664 × 10^+0^	3.046 × 10^+0^	3.263 × 10^+0^	2.660 × 10^+0^	4.601 × 10^+0^	4.401 × 10^+0^	3.959 × 10^+0^	2.824 × 10^+0^	**2.422 × 10^+0^**
	Mean	4.593 × 10^+0^	4.674 × 10^+0^	4.015 × 10^+0^	4.329 × 10^+0^	4.187 × 10^+0^	4.238 × 10^+0^	3.980 × 10^+0^	4.967 × 10^+0^	4.757 × 10^+0^	4.508 × 10^+0^	3.718 × 10^+0^	**3.346 × 10^+0^**
	Std	1.869 × 10^−1^	**1.115 × 10^−1^**	4.525 × 10^−1^	3.270 × 10^−1^	4.710 × 10^−1^	3.805 × 10^−1^	3.995 × 10^−1^	1.828 × 10^−1^	1.824 × 10^−1^	2.327 × 10^−1^	5.252 × 10^−1^	4.517 × 10^−1^
	Rank	9	10	4	7	6	5	3	12	11	8	2	1
F9	Best	1.630 × 10^+0^	1.545 × 10^+0^	1.120 × 10^+0^	1.098 × 10^+0^	1.071 × 10^+0^	1.174 × 10^+0^	1.167 × 10^+0^	1.101 × 10^+0^	1.783 × 10^+0^	1.129 × 10^+0^	1.055 × 10^+0^	**1.052 × 10^+0^**
	Mean	1.914 × 10^+0^	1.757 × 10^+0^	1.233 × 10^+0^	1.281 × 10^+0^	1.216 × 10^+0^	1.316 × 10^+0^	1.351 × 10^+0^	1.220 × 10^+0^	3.543 × 10^+0^	1.279 × 10^+0^	**1.153 × 10^+0^**	1.160 × 10^+0^
	Std	2.040 × 10^−1^	1.279 × 10^−1^	6.429 × 10^−2^	1.155 × 10^−1^	9.194 × 10^−2^	1.058 × 10^−1^	1.484 × 10^−1^	9.284 × 10^−2^	5.414 × 10^−1^	6.533 × 10^−2^	6.095 × 10^−2^	**4.296 × 10^−2^**
	Rank	11	10	5	6	4	8	9	3	12	7	1	2
F10	Best	2.130 × 10^+1^	2.118 × 10^+1^	2.138 × 10^+1^	2.100 × 10^+1^	2.113 × 10^+1^	**3.182 × 10^+0^**	2.122 × 10^+1^	2.125 × 10^+1^	2.115 × 10^+1^	2.117 × 10^+1^	2.101 × 10^+1^	2.066 × 10^+1^
	Mean	2.154 × 10^+1^	2.127 × 10^+1^	2.155 × 10^+1^	2.113 × 10^+1^	2.148 × 10^+1^	**2.060 × 10^+1^**	2.143 × 10^+1^	2.151 × 10^+1^	2.130 × 10^+1^	2.135 × 10^+1^	2.102 × 10^+1^	2.109 × 10^+1^
	Std	8.603 × 10^−2^	4.953 × 10^−2^	7.986 × 10^−2^	1.344 × 10^−1^	1.438 × 10^−1^	3.306 × 10^+0^	9.677 × 10^−2^	1.089 × 10^−1^	6.870 × 10^−2^	9.977 × 10^−2^	**9.181 × 10^−3^**	9.259 × 10^−2^
	Rank	11	5	12	3	9	4	8	10	6	7	1	2
Mean Rank		10.3	9.1	5.7	6.3	4.5	5.3	6	6.9	10.4	7.9	3.4	**2.2**
+/=/−		11/1/0	11/0/1	10/1/1	10/1/1	9/1/2	8/0/4	10/2/0	10/0/2	12/0/0	12/0/0	9/2/1	~

**Table 8:** Path planning results in simple environments.

Map	Index	PSO	GWO	NGO	GTO	BBO	DMBBO
1	Best	2.980 × 10^+1^	3.038 × 10^+1^	3.121 × 10^+1^	3.180 × 10^+1^	**2.921 × 10^+1^**	**2.921 × 10^+1^**
	Mean	3.236 × 10^+1^	3.283 × 10^+1^	3.246 × 10^+1^	3.262 × 10^+1^	3.260 × 10^+1^	**3.186 × 10^+1^**
	Std	1.490 × 10^+0^	1.174 × 10^+0^	4.278 × 10^−1^	**4.241 × 10^−1^**	1.472 × 10^+0^	1.605 × 10^+0^
	Rank	2	5	4	3	6	1
2	Best	4.570 × 10^+1^	4.594 × 10^+1^	4.711 × 10^+1^	4.653 × 10^+1^	4.453 × 10^+1^	**4.336 × 10^+1^**
	Mean	4.864 × 10^+1^	4.777 × 10^+1^	4.883 × 10^+1^	5.061 × 10^+1^	4.790 × 10^+1^	**4.678 × 10^+1^**
	Std	1.762 × 10^+0^	2.204 × 10^+0^	**7.981 × 10^−1^**	2.020 × 10^+0^	1.656 × 10^+0^	1.748 × 10^+0^
	Rank	4	2	5	6	3	1
3	Best	6.125 × 10^+1^	5.925 × 10^+1^	6.125 × 10^+1^	5.925 × 10^+1^	**5.808 × 10^+1^**	**5.808 × 10^+1^**
	Mean	6.382 × 10^+1^	6.256 × 10^+1^	6.182 × 10^+1^	**6.033 × 10^+1^**	6.337 × 10^+1^	6.103 × 10^+1^
	Std	2.974 × 10^+0^	3.008 × 10^+0^	**1.069 × 10^−1^**	6.942 × 10^−1^	3.413 × 10^+0^	2.275 × 10^+0^
	Rank	6	3	4	1	5	2
Mean Rank		4	3.3	4.3	3.3	4.7	**1.3**
Final Ranking		4	2	5	2	6	1

**Table 9:** Path planning results in complex environments.

Map	Index	PSO	GWO	NGO	GTO	BBO	DMBBO
4	Best	**3.121 × 10^+1^**	**3.121 × 10^+1^**	3.180 × 10^+1^	3.238 × 10^+1^	**3.121 × 10^+1^**	**3.121 × 10^+1^**
	Mean	3.362 × 10^+1^	3.478 × 10^+1^	3.400 × 10^+1^	3.507 × 10^+1^	3.220 × 10^+1^	**3.174 × 10^+1^**
	Std	1.652 × 10^+0^	2.867 × 10^+0^	1.798 × 10^+0^	2.030 × 10^+0^	1.146 × 10^+0^	**5.930 × 10^−1^**
	Rank	3	4	5	6	2	1
5	Best	4.853 × 10^+1^	4.853 × 10^+1^	4.853 × 10^+1^	4.911 × 10^+1^	4.853 × 10^+1^	**4.794 × 10^+1^**
	Mean	5.081 × 10^+1^	5.212 × 10^+1^	5.200 × 10^+1^	5.413 × 10^+1^	5.067 × 10^+1^	**5.058 × 10^+1^**
	Std	1.884 × 10^+0^	2.672 × 10^+0^	1.887 × 10^+0^	3.231 × 10^+0^	**1.865 × 10^+0^**	2.119 × 10^+0^
	Rank	2	4	5	6	3	1
6	Best	6.160 × 10^+1^	6.160 × 10^+1^	6.501 × 10^+1^	6.677 × 10^+1^	6.184 × 10^+1^	**5.984 × 10^+1^**
	Mean	6.777 × 10^+1^	6.758 × 10^+1^	6.880 × 10^+1^	7.114 × 10^+1^	6.585 × 10^+1^	**6.485 × 10^+1^**
	Std	3.162 × 10^+0^	3.191 × 10^+0^	**1.459 × 10^+0^**	2.408 × 10^+0^	2.934 × 10^+0^	2.681 × 10^+0^
	Rank	3	4	5	6	2	1
Mean Rank		2.7	4	5	6	2.3	**1**
Final Ranking		3	4	5	6	2	1

## Data Availability

The data used to support the findings of this study are included in the article.

## Appendix A

### Appendix A.1

Time Complexity of DMBBO:

When the population is divided into K niches, the time complexity of DMBBO is $O({iter}_{max}\cdot N\cdot (log(N/K)+dim+f(\cdot )))$, where ${iter}_{max}$ is the maximum iteration count.

The additional overhead introduced by DMBBO includes:

- Multi-niche division and sorting initialization: Dividing the population into K niches and initial sorting requires O(N · log(N)). This occurs only during the initialization phase and is therefore negligible.
- Diversity calculation for dual-source migration: Pre-calculating spatial and fitness diversity indices for all individuals in each niche requires O(K · (N/K)2 · dim) = O(N2 · dim/K). With K=3, the complexity per iteration is O(N2 · dim/3). Since this is amortized over N individuals and T iterations, the effective overhead is O(N · dim) per individual, which is comparable to the position update cost.
- Intra-niche sorting: Sorting individuals within each niche after migration has complexity O(K · (N/K) · log(N/K)) = O(N · log(N/K)). With K=3, this simplifies to O(N · log(N)) per iteration, which is negligible compared to function evaluations.
- Adaptive dual-preservation strategy: Selecting elite and diversity individuals from the top 50% within each niche requires O(K · (N/2K) · dim) = O(N · dim/2). The additional overhead is minimal.
- Inter-niche elite migration: With $\lambda_{out}\;=\;0.1$, only approximately 10% of dimension updates involve inter-niche migration, contributing O(0.1 · N · dim) per iteration, which is negligible.
  Therefore, with K=3 and $\lambda_{out}=0.1$, the overall time complexity of DMBBO is:
  $$O(N\;\cdot \;(dim\;+\;f(\cdot )))\;+\;O({iter}_{max}\;\cdot \;N\;\cdot \;(log(N/K)\;+\;(1\;+\;N/K)\;\cdot \;dim\;+\;f(\cdot )))\;=\;O({iter}_{max}\cdot \;N\;\cdot \;(log(N)\;+\;N\;\cdot \;dim/3\;+\;f(\cdot )))$$
  Since K=3 is a small constant and the diversity calculation overhead O(N · dim/3) is proportional to N, the simplified form is:
  $$O({iter}_{max}\cdot \;N\;\cdot \;(log(N)\;+\;N\;\cdot \;dim\;+\;f(\cdot )))$$

The dominant terms are the diversity calculation overhead O(N2 · dim) periteration and the function evaluation cost O(N · f(·)) per iteration, making DMBBO slightly more expensive than standard BBO in terms of computational overhead, but this is offset by improved convergence speed and solution quality. Therefore, the proposed improvements enhance search performance without significantly increasing computational complexity.

### Appendix A.2

**Table A1:** CEC2022 benchmark functions.

Type	No.	Functions	$F_{i}^{*}=F_{i}(X^{*})$
Unimodal Functions	1	Shifted and full Rotated Zakharov Function	300
Basic Functions	2	Shifted and full Rotated Rosenbrock’s Function	400
	3	Shifted and full Rotated Expanded Schaffer’s f6 Function	600
	4	Shifted and full Rotated Non-Continuous Rastrigin’s Function	800
	5	Shifted and full Rotated Levy Function	900
Hybrid Functions	6	Hybrid Function 1 (N = 3)	1800
	7	Hybrid Function 2 (N = 3)	2000
	8	Hybrid Function 3 (N = 3)	2200
Composition Functions	9	Composition Function 1 (N = 3)	2300
	10	Composition Function 2 (N = 4)	2400
	11	Composition Function 3 (N = 5)	2600
	12	Composition Function 4 (N = 6)	2700
Search Range: ${\left[-100,100\right]}^{D}$			

**Table A2:** CEC2020 benchmark functions.

Type	No.	Functions	$F_{i}^{*}=F_{i}(X^{*})$
Unimodal Functions	1	Shifted and Rotated Bent Cigar Function	100
Basic Functions	2	Shifted and Rotated Schwefel’s Function	1100
	3	Shifted and Rotated Rastrigin’s Function	700
	4	Shifted and Rotated Lunacek bi-Rastrigin Function	1900
	5	Hybrid Function 1 (N = 3)	1700
Hybrid Functions	6	Hybrid Function 2 (N = 3)	1600
	7	Hybrid Function 3 (N = 3)	2100
Composition Functions	8	Composition Function 1 (N = 3)	2200
	9	Composition Function 2 (N = 4)	2400
	10	Composition Function 3 (N = 5)	2500
Search Range: ${\left[-100,100\right]}^{D}$			

**Table A3:** CEC2019 benchmark functions.

No.	Functions	$F_{i}^{*}=F_{i}(X^{*})$	D	Search Range
1	Storn’s Chebyshev Polynomial Fitting Problem	1	9	[−8192, 8192]
2	Inverse Hilbert Matrix Problem	1	16	[−16,384, 16,384]
3	Lennard-Jones Minimum Energy Cluster	1	18	[−4, 4]
4	Rastrigin’s Function	1	10	[−100, 100]
5	Griewangk’s Function	1	10	[−100, 100]
6	Weierstrass Function	1	10	[−100, 100]
7	Modified Schwefel’s Function	1	10	[−100, 100]
8	Expanded Schaffer’s F6 Function	1	10	[−100, 100]
9	Happy Cat Function	1	10	[−100, 100]
10	Ackley Function	1	10	[−100, 100]

**Table A4:** Sensitivity analysis results for parameter K on CEC2022 (Dim = 10).

Function	Item Name	K				
		2	3	4	5	6
F1	Best	3.000 × 10^+2^	3.001 × 10^+2^	3.003 × 10^+2^	3.000 × 10^+2^	3.009 × 10^+2^
	Mean	3.022 × 10^+2^	3.067 × 10^+2^	3.151 × 10^+2^	3.714 × 10^+2^	4.143 × 10^+2^
	Std	2.195 × 10^+0^	2.015 × 10^+1^	2.637 × 10^+1^	1.985 × 10^+2^	2.270 × 10^+2^
	Rank	1	2	3	4	5
F2	Best	4.000 × 10^+2^	4.000 × 10^+2^	4.000 × 10^+2^	4.000 × 10^+2^	4.000 × 10^+2^
	Mean	4.026 × 10^+2^	4.041 × 10^+2^	4.010 × 10^+2^	4.025 × 10^+2^	4.023 × 10^+2^
	Std	2.713 × 10^+0^	1.204 × 10^+1^	1.826 × 10^+0^	9.706 × 10^+0^	1.039 × 10^+1^
	Rank	4	5	1	3	2
F3	Best	6.000 × 10^+2^	6.000 × 10^+2^	6.000 × 10^+2^	6.000 × 10^+2^	6.000 × 10^+2^
	Mean	6.000 × 10^+2^	6.000 × 10^+2^	6.000 × 10^+2^	6.000 × 10^+2^	6.000 × 10^+2^
	Std	1.021 × 10^−2^	6.588 × 10^−3^	7.508 × 10^−3^	1.879 × 10^−3^	8.000 × 10^−3^
	Rank	5	4	3	1	2
F4	Best	8.020 × 10^+2^	8.030 × 10^+2^	8.010 × 10^+2^	8.030 × 10^+2^	8.020 × 10^+2^
	Mean	8.090 × 10^+2^	8.078 × 10^+2^	8.077 × 10^+2^	8.060 × 10^+2^	8.067 × 10^+2^
	Std	3.980 × 10^+0^	3.074 × 10^+0^	3.231 × 10^+0^	2.133 × 10^+0^	3.067 × 10^+0^
	Rank	5	4	3	1	2
F5	Best	9.000 × 10^+2^	9.000 × 10^+2^	9.000 × 10^+2^	9.000 × 10^+2^	9.000 × 10^+2^
	Mean	9.000 × 10^+2^	9.000 × 10^+2^	9.000 × 10^+2^	9.000 × 10^+2^	9.000 × 10^+2^
	Std	2.272 × 10^−2^	2.448 × 10^−2^	4.803 × 10^−3^	2.212 × 10^−3^	9.714 × 10^−3^
	Rank	4	5	2	1	3
F6	Best	1.828 × 10^+3^	1.816 × 10^+3^	1.824 × 10^+3^	1.835 × 10^+3^	1.824 × 10^+3^
	Mean	2.875 × 10^+3^	2.540 × 10^+3^	2.304 × 10^+3^	2.247 × 10^+3^	2.053 × 10^+3^
	Std	1.459 × 10^+3^	1.143 × 10^+3^	8.652 × 10^+2^	6.337 × 10^+2^	2.178 × 10^+2^
	Rank	5	4	3	2	1
F7	Best	2.000 × 10^+3^	2.000 × 10^+3^	2.000 × 10^+3^	2.001 × 10^+3^	2.000 × 10^+3^
	Mean	2.013 × 10^+3^	2.013 × 10^+3^	2.009 × 10^+3^	2.009 × 10^+3^	2.008 × 10^+3^
	Std	1.349 × 10^+1^	1.339 × 10^+1^	1.049 × 10^+1^	9.897 × 10^+0^	8.867 × 10^+0^
	Rank	4	5	2	3	1
F8	Best	2.200 × 10^+3^	2.200 × 10^+3^	2.201 × 10^+3^	2.200 × 10^+3^	2.200 × 10^+3^
	Mean	2.217 × 10^+3^	2.218 × 10^+3^	2.216 × 10^+3^	2.217 × 10^+3^	2.217 × 10^+3^
	Std	7.484 × 10^+0^	6.407 × 10^+0^	7.313 × 10^+0^	6.947 × 10^+0^	6.487 × 10^+0^
	Rank	4	5	1	2	3
F9	Best	2.529 × 10^+3^	2.529 × 10^+3^	2.529 × 10^+3^	2.529 × 10^+3^	2.529 × 10^+3^
	Mean	2.529 × 10^+3^	2.529 × 10^+3^	2.529 × 10^+3^	2.530 × 10^+3^	2.530 × 10^+3^
	Std	1.006 × 10^−1^	1.062 × 10^−1^	3.354 × 10^−1^	1.753 × 10^+0^	1.575 × 10^+0^
	Rank	1	2	3	4	5
F10	Best	2.500 × 10^+3^	2.500 × 10^+3^	2.500 × 10^+3^	2.500 × 10^+3^	2.412 × 10^+3^
	Mean	2.511 × 10^+3^	2.519 × 10^+3^	2.515 × 10^+3^	2.508 × 10^+3^	2.505 × 10^+3^
	Std	3.341 × 10^+1^	4.166 × 10^+1^	3.694 × 10^+1^	2.751 × 10^+1^	3.238 × 10^+1^
	Rank	3	5	4	2	1
F11	Best	2.600 × 10^+3^	2.600 × 10^+3^	2.600 × 10^+3^	2.600 × 10^+3^	2.600 × 10^+3^
	Mean	2.620 × 10^+3^	2.610 × 10^+3^	2.615 × 10^+3^	2.601 × 10^+3^	2.600 × 10^+3^
	Std	6.523 × 10^+1^	3.814 × 10^+1^	6.036 × 10^+1^	2.685 × 10^+0^	7.667 × 10^−1^
	Rank	5	3	4	2	1
F12	Best	2.865 × 10^+3^	2.865 × 10^+3^	2.865 × 10^+3^	2.865 × 10^+3^	2.864 × 10^+3^
	Mean	2.870 × 10^+3^	2.869 × 10^+3^	2.869 × 10^+3^	2.869 × 10^+3^	2.869 × 10^+3^
	Std	3.660 × 10^+0^	2.352 × 10^+0^	3.682 × 10^+0^	2.449 × 10^+0^	4.624 × 10^+0^
	Rank	5	3	4	1	2
Mean Rank		3.8	3.9	2.7	2.2	2.3

**Table A5:** Sensitivity analysis results for parameter $\lambda_{out}$ on CEC2022 (dim = 10).

Function	Item Name	$\lambda_{out}$				
		0.05	0.1	0.15	0.2	0.25
F1	Best	3.003 × 10^+2^	3.000 × 10^+2^	3.000 × 10^+2^	3.001 × 10^+2^	3.001 × 10^+2^
	Mean	3.332 × 10^+2^	3.111 × 10^+2^	3.023 × 10^+2^	3.024 × 10^+2^	3.028 × 10^+2^
	Std	4.932 × 10^+1^	2.567 × 10^+1^	2.587 × 10^+0^	3.689 × 10^+0^	3.281 × 10^+0^
	Rank	5	4	1	2	3
F2	Best	4.000 × 10^+2^	4.000 × 10^+2^	4.000 × 10^+2^	4.000 × 10^+2^	4.000 × 10^+2^
	Mean	4.012 × 10^+2^	4.014 × 10^+2^	4.023 × 10^+2^	4.069 × 10^+2^	4.037 × 10^+2^
	Std	1.771 × 10^+0^	1.931 × 10^+0^	2.635 × 10^+0^	1.685 × 10^+1^	3.364 × 10^+0^
	Rank	1	2	3	5	4
F3	Best	6.000 × 10^+2^	6.000 × 10^+2^	6.000 × 10^+2^	6.000 × 10^+2^	6.000 × 10^+2^
	Mean	6.000 × 10^+2^	6.000 × 10^+2^	6.000 × 10^+2^	6.000 × 10^+2^	6.000 × 10^+2^
	Std	5.961 × 10^−3^	4.332 × 10^−3^	9.084 × 10^−3^	9.584 × 10^−3^	1.064 × 10^−2^
	Rank	2	1	3	4	5
F4	Best	8.050 × 10^+2^	8.020 × 10^+2^	8.030 × 10^+2^	8.011 × 10^+2^	8.030 × 10^+2^
	Mean	8.093 × 10^+2^	8.078 × 10^+2^	8.074 × 10^+2^	8.064 × 10^+2^	8.080 × 10^+2^
	Std	3.194 × 10^+0^	2.914 × 10^+0^	3.362 × 10^+0^	2.748 × 10^+0^	2.858 × 10^+0^
	Rank	5	3	2	1	4
F5	Best	9.000 × 10^+2^	9.000 × 10^+2^	9.000 × 10^+2^	9.000 × 10^+2^	9.000 × 10^+2^
	Mean	9.000 × 10^+2^	9.000 × 10^+2^	9.000 × 10^+2^	9.000 × 10^+2^	9.000 × 10^+2^
	Std	2.477 × 10^−2^	1.630 × 10^−2^	1.625 × 10^−2^	1.699 × 10^−2^	8.925 × 10^−2^
	Rank	4	3	2	1	5
F6	Best	1.825 × 10^+3^	1.831 × 10^+3^	1.823 × 10^+3^	1.860 × 10^+3^	1.831 × 10^+3^
	Mean	2.059 × 10^+3^	2.246 × 10^+3^	2.262 × 10^+3^	3.013 × 10^+3^	3.529 × 10^+3^
	Std	3.055 × 10^+2^	6.732 × 10^+2^	4.965 × 10^+2^	1.213 × 10^+3^	1.499 × 10^+3^
	Rank	1	2	3	4	5
F7	Best	2.000 × 10^+3^	2.000 × 10^+3^	2.000 × 10^+3^	2.000 × 10^+3^	2.000 × 10^+3^
	Mean	2.010 × 10^+3^	2.009 × 10^+3^	2.012 × 10^+3^	2.013 × 10^+3^	2.010 × 10^+3^
	Std	1.015 × 10^+1^	9.793 × 10^+0^	1.017 × 10^+1^	1.028 × 10^+1^	1.028 × 10^+1^
	Rank	3	1	4	5	2
F8	Best	2.202 × 10^+3^	2.203 × 10^+3^	2.201 × 10^+3^	2.201 × 10^+3^	2.201 × 10^+3^
	Mean	2.219 × 10^+3^	2.219 × 10^+3^	2.219 × 10^+3^	2.218 × 10^+3^	2.219 × 10^+3^
	Std	4.536 × 10^+0^	4.526 × 10^+0^	4.945 × 10^+0^	6.470 × 10^+0^	5.784 × 10^+0^
	Rank	5	3	4	1	2
F9	Best	2.529 × 10^+3^	2.529 × 10^+3^	2.529 × 10^+3^	2.529 × 10^+3^	2.529 × 10^+3^
	Mean	2.530 × 10^+3^	2.529 × 10^+3^	2.529 × 10^+3^	2.529 × 10^+3^	2.529 × 10^+3^
	Std	5.522 × 10^−1^	2.813 × 10^−1^	6.032 × 10^−1^	4.349 × 10^−2^	1.573 × 10^−1^
	Rank	5	3	4	1	2
F10	Best	2.500 × 10^+3^	2.500 × 10^+3^	2.500 × 10^+3^	2.500 × 10^+3^	2.500 × 10^+3^
	Mean	2.508 × 10^+3^	2.504 × 10^+3^	2.530 × 10^+3^	2.519 × 10^+3^	2.545 × 10^+3^
	Std	2.736 × 10^+1^	1.994 × 10^+1^	5.028 × 10^+1^	4.142 × 10^+1^	5.591 × 10^+1^
	Rank	2	1	4	3	5
F11	Best	2.600 × 10^+3^	2.600 × 10^+3^	2.600 × 10^+3^	2.600 × 10^+3^	2.600 × 10^+3^
	Mean	2.605 × 10^+3^	2.620 × 10^+3^	2.620 × 10^+3^	2.615 × 10^+3^	2.643 × 10^+3^
	Std	2.744 × 10^+1^	6.519 × 10^+1^	6.519 × 10^+1^	6.043 × 10^+1^	9.896 × 10^+1^
	Rank	1	3	4	2	5
F12	Best	2.865 × 10^+3^	2.865 × 10^+3^	2.865 × 10^+3^	2.865 × 10^+3^	2.867 × 10^+3^
	Mean	2.868 × 10^+3^	2.869 × 10^+3^	2.868 × 10^+3^	2.870 × 10^+3^	2.870 × 10^+3^
	Std	1.850 × 10^+0^	4.526 × 10^+0^	1.794 × 10^+0^	3.926 × 10^+0^	3.522 × 10^+0^
	Rank	1	3	2	4	5
Mean Rank		2.9	2.4	3	2.8	3.9

**Table A6:** Sensitivity analysis results for parameter $t_{dp}$ on CEC2022 (dim = 10).

Function	Item Name	$t_{dp}$				
		0.1	0.2	0.3	0.4	0.5
F1	Best	3.001 × 10^+2^	3.001 × 10^+2^	3.001 × 10^+2^	3.000 × 10^+2^	3.000 × 10^+2^
	Mean	3.028 × 10^+2^	3.057 × 10^+2^	3.054 × 10^+2^	3.043 × 10^+2^	3.057 × 10^+2^
	Std	4.931 × 10^+0^	9.214 × 10^+0^	1.188 × 10^+1^	6.215 × 10^+0^	9.023 × 10^+0^
	Rank	1	4	3	2	5
F2	Best	4.000 × 10^+2^	4.000 × 10^+2^	4.000 × 10^+2^	4.000 × 10^+2^	4.000 × 10^+2^
	Mean	4.013 × 10^+2^	4.015 × 10^+2^	4.011 × 10^+2^	4.016 × 10^+2^	4.016 × 10^+2^
	Std	2.218 × 10^+0^	2.291 × 10^+0^	2.075 × 10^+0^	2.449 × 10^+0^	2.502 × 10^+0^
	Rank	2	3	1	5	4
F3	Best	6.000 × 10^+2^	6.000 × 10^+2^	6.000 × 10^+2^	6.000 × 10^+2^	6.000 × 10^+2^
	Mean	6.000 × 10^+2^	6.000 × 10^+2^	6.000 × 10^+2^	6.000 × 10^+2^	6.000 × 10^+2^
	Std	7.237 × 10^−3^	5.863 × 10^−3^	5.586 × 10^−3^	1.100 × 10^−2^	6.947 × 10^−3^
	Rank	3	2	1	5	4
F4	Best	8.030 × 10^+2^	8.030 × 10^+2^	8.020 × 10^+2^	8.040 × 10^+2^	8.030 × 10^+2^
	Mean	8.084 × 10^+2^	8.089 × 10^+2^	8.073 × 10^+2^	8.088 × 10^+2^	8.077 × 10^+2^
	Std	3.589 × 10^+0^	3.502 × 10^+0^	3.118 × 10^+0^	3.323 × 10^+0^	2.677 × 10^+0^
	Rank	3	5	1	4	2
F5	Best	9.000 × 10^+2^	9.000 × 10^+2^	9.000 × 10^+2^	9.000 × 10^+2^	9.000 × 10^+2^
	Mean	9.000 × 10^+2^	9.000 × 10^+2^	9.000 × 10^+2^	9.000 × 10^+2^	9.000 × 10^+2^
	Std	4.283 × 10^−3^	2.736 × 10^−2^	2.269 × 10^−2^	2.729 × 10^−2^	3.097 × 10^−2^
	Rank	1	4	2	3	5
F6	Best	1.815 × 10^+3^	1.824 × 10^+3^	1.827 × 10^+3^	1.827 × 10^+3^	1.824 × 10^+3^
	Mean	2.571 × 10^+3^	2.958 × 10^+3^	2.252 × 10^+3^	2.354 × 10^+3^	2.328 × 10^+3^
	Std	7.899 × 10^+2^	1.358 × 10^+3^	5.179 × 10^+2^	6.272 × 10^+2^	7.262 × 10^+2^
	Rank	4	5	1	3	2
F7	Best	2.000 × 10^+3^	2.000 × 10^+3^	2.000 × 10^+3^	2.000 × 10^+3^	2.000 × 10^+3^
	Mean	2.012 × 10^+3^	2.009 × 10^+3^	2.013 × 10^+3^	2.011 × 10^+3^	2.012 × 10^+3^
	Std	1.247 × 10^+1^	9.680 × 10^+0^	1.318 × 10^+1^	1.031 × 10^+1^	9.845 × 10^+0^
	Rank	4	1	5	2	3
F8	Best	2.200 × 10^+3^	2.200 × 10^+3^	2.201 × 10^+3^	2.200 × 10^+3^	2.201 × 10^+3^
	Mean	2.218 × 10^+3^	2.219 × 10^+3^	2.217 × 10^+3^	2.218 × 10^+3^	2.217 × 10^+3^
	Std	5.774 × 10^+0^	4.717 × 10^+0^	7.373 × 10^+0^	6.014 × 10^+0^	7.278 × 10^+0^
	Rank	4	5	1	3	2
F9	Best	2.529 × 10^+3^	2.529 × 10^+3^	2.529 × 10^+3^	2.529 × 10^+3^	2.529 × 10^+3^
	Mean	2.529 × 10^+3^	2.529 × 10^+3^	2.529 × 10^+3^	2.529 × 10^+3^	2.529 × 10^+3^
	Std	4.626 × 10^−1^	6.619 × 10^−1^	4.757 × 10^−1^	7.026 × 10^−3^	1.144 × 10^−1^
	Rank	3	5	4	1	2
F10	Best	2.500 × 10^+3^	2.500 × 10^+3^	2.500 × 10^+3^	2.500 × 10^+3^	2.500 × 10^+3^
	Mean	2.515 × 10^+3^	2.511 × 10^+3^	2.529 × 10^+3^	2.519 × 10^+3^	2.508 × 10^+3^
	Std	3.826 × 10^+1^	3.332 × 10^+1^	4.888 × 10^+1^	4.247 × 10^+1^	2.777 × 10^+1^
	Rank	3	2	5	4	1
F11	Best	2.600 × 10^+3^	2.600 × 10^+3^	2.600 × 10^+3^	2.600 × 10^+3^	2.600 × 10^+3^
	Mean	2.657 × 10^+3^	2.625 × 10^+3^	2.610 × 10^+3^	2.620 × 10^+3^	2.615 × 10^+3^
	Std	1.181 × 10^+2^	5.694 × 10^+1^	3.810 × 10^+1^	5.199 × 10^+1^	6.038 × 10^+1^
	Rank	5	4	1	3	2
F12	Best	2.865 × 10^+3^	2.865 × 10^+3^	2.865 × 10^+3^	2.865 × 10^+3^	2.865 × 10^+3^
	Mean	2.870 × 10^+3^	2.869 × 10^+3^	2.869 × 10^+3^	2.870 × 10^+3^	2.870 × 10^+3^
	Std	3.266 × 10^+0^	3.336 × 10^+0^	4.179 × 10^+0^	4.604 × 10^+0^	5.519 × 10^+0^
	Rank	4	2	1	5	3
Mean Rank		3.1	3.5	2.2	3.3	2.9
